# Laboratory evidence of dynamo amplification of magnetic fields in a turbulent plasma

**DOI:** 10.1038/s41467-018-02953-2

**Published:** 2018-02-09

**Authors:** P. Tzeferacos, A. Rigby, A. F. A. Bott, A. R. Bell, R. Bingham, A. Casner, F. Cattaneo, E. M. Churazov, J. Emig, F. Fiuza, C. B. Forest, J. Foster, C. Graziani, J. Katz, M. Koenig, C.-K. Li, J. Meinecke, R. Petrasso, H.-S. Park, B. A. Remington, J. S. Ross, D. Ryu, D. Ryutov, T. G. White, B. Reville, F. Miniati, A. A. Schekochihin, D. Q. Lamb, D. H. Froula, G. Gregori

**Affiliations:** 1 Department of Physics, University of Oxford, Parks Road, Oxford, OX1 3PU UK; 2 Department of Astronomy and Astrophysics, University of Chicago, 5640 S. Ellis Ave, Chicago, IL 60637 USA; 3 Rutherford Appleton Laboratory, Chilton, Didcot, OX11 0QX UK; 4 Department of Physics, University of Strathclyde, Glasgow, G4 0NG UK; 5 CEA, DAM, DIF, 91297 Arpajon, France; 6 Max Planck Institute for Astrophysics, Karl-Schwarzschild-Strasse 1, 85741 Garching, Germany; 7 Space Research Institute (IKI), Profsouznaya 84/32, Moscow, 117997 Russia; 8 Lawrence Livermore National Laboratory, Livermore, CA 94550 USA; 9 SLAC National Accelerator Laboratory, 2575 Sand Hill Road, Menlo Park, CA 94025 USA; 10 Physics Department, University of Wisconsin-Madison, 1150 University Avenue, Madison, WI 53706 USA; 11 AWE, Aldermaston, Reading, West Berkshire, RG7 4PR UK; 12 Laboratory for Laser Energetics, University of Rochester, 250 E. River Rd, Rochester, NY 14623 USA; 13 Laboratoire pour l’Utilisation de Lasers Intenses, UMR7605, CNRS CEA, Université Paris VI Ecole Polytechnique, 91128 Palaiseau Cedex, France; 14 Massachusetts Institute of Technology, Cambridge, MA 02139 USA; 15 Department of Physics, UNIST, Ulsan, 689-798 Korea; 16 School of Mathematics and Physics, Queens University Belfast, Belfast, BT7 1NN UK; 17 Department of Physics, ETH Zürich, Wolfgang-Pauli-Strasse 27, Zürich, CH-8093 Switzerland

## Abstract

Magnetic fields are ubiquitous in the Universe. The energy density of these fields is typically comparable to the energy density of the fluid motions of the plasma in which they are embedded, making magnetic fields essential players in the dynamics of the luminous matter. The standard theoretical model for the origin of these strong magnetic fields is through the amplification of tiny seed fields via turbulent dynamo to the level consistent with current observations. However, experimental demonstration of the turbulent dynamo mechanism has remained elusive, since it requires plasma conditions that are extremely hard to re-create in terrestrial laboratories. Here we demonstrate, using laser-produced colliding plasma flows, that turbulence is indeed capable of rapidly amplifying seed fields to near equipartition with the turbulent fluid motions. These results support the notion that turbulent dynamo is a viable mechanism responsible for the observed present-day magnetization.

## Abstract (web summary)

Exploring astrophysical turbulent effects in laboratory plasma is challenging due to high threshold values of relevant parameters, such as the magnetic Reynolds number. Here the authors demonstrate the turbulent dynamo effect at large magnetic Reynolds numbers in laser-generated magnetized plasma.

## Introduction

Diffuse radio-synchrotron emission observations and Faraday rotation measurements^1^ have revealed magnetic field strengths ranging from a few nG and tens of μG in extragalactic disks, halos and clusters, up to hundreds of TG in magnetars, as inferred from their spin-down^2^. That turbulence is of central importance in the generation and evolution of magnetic fields in the Universe is widely accepted^3^. Plasma turbulence can be found in myriads of astrophysical objects, where it is excited by a range of processes: cluster mergers, supernovae explosions, stellar outflows, etc.^4–7^. If a turbulent plasma is threaded by a weak magnetic field, the stochastic motions of the fluid will stretch and fold this field, amplifying it until it becomes dynamically significant^8,9^. According to the current standard picture, the amplification can be summarized in two basic steps^10,11^. First, when the initial field is small the magnetic energy grows exponentially (kinematic phase). This phase terminates when the magnetic energy reaches approximate equipartition with the kinetic energy at the dissipation scale. Beyond this point, the magnetic energy continues to grow linearly in time (nonlinear phase) until, after roughly one outer-scale eddy-turnover time, it saturates at a fraction of the total kinetic energy of the fluid motions^10,12^. This is what is referred to as the turbulent dynamo mechanism for magnetic field amplification.

The seed fields that the dynamo amplifies can be produced by a variety of different physical processes. In many astrophysical environments where the plasma is initially unmagnetized, and most certainly at the time when proto-galaxies were forming, baroclinic generation of magnetic fields due to misaligned density and temperature gradients—the Biermann battery mechanism—can provide initial seeds^13^. The same starting fields also occur in laser-produced plasmas^14,15^.

Theoretical expectations that turbulent dynamo must operate go back more than half a century^8,9,16^ and the first direct numerical confirmation of this effect was achieved 35 years ago^17^. A significant body of theoretical work has developed over the years^18–20^ that has greatly expanded our understanding of the mechanism—for recent reviews of the current state of affairs see refs. ^21 and ^^22^. Despite these advancements, demonstrating turbulent dynamo amplification in the laboratory has remained elusive. This is primarily because of the difficulty of achieving experimentally magnetic Reynolds numbers (Rm = *u*_*L*_*L*/*μ*, where *u*_*L*_ is the flow velocity at the outer scale *L*, and *μ* is the magnetic diffusivity) above the critical threshold of a few hundred required for dynamo^23^. Such a demonstration would not only establish experimentally the soundness of the existing theoretical and numerical expectations for one of the most fundamental physical processes in astrophysics^24^, but also provide a platform^25^ to investigate other fundamental processes that require a turbulent magnetized plasma, such as particle acceleration and reconnection.

To date, experimental investigation of magnetic field amplification has primarily been carried out in liquid-metal experiments, such as the von Kármán swirling flow of ref. ^26^ and the Riga dynamo experiment^27,28^ inspired by the Ponomarenko dynamo^29^. The driven dynamos achieved in these experiments depended on a particular fluid flow rather than a purely turbulent effect leading to a stochastic field. More recent work has focused on laser-driven plasmas^25,30,31^, but studying a regime that is a precursor to dynamo, because of the modest magnetic Reynolds numbers that could be achieved.

In the following, we describe experiments in which we reach magnetic Reynolds numbers above the expected dynamo threshold. We detail the experimental configuration and present diagnostic measurements that fully characterize the plasma state of the magnetized turbulence. By utilizing two independent magnetic field diagnostics we are able to demonstrate the turbulent dynamo amplification of seed magnetic fields to dynamical equipartition with the kinetic energy of the turbulent motions.

## Results

### Experimental platform

**Fig. 1:**
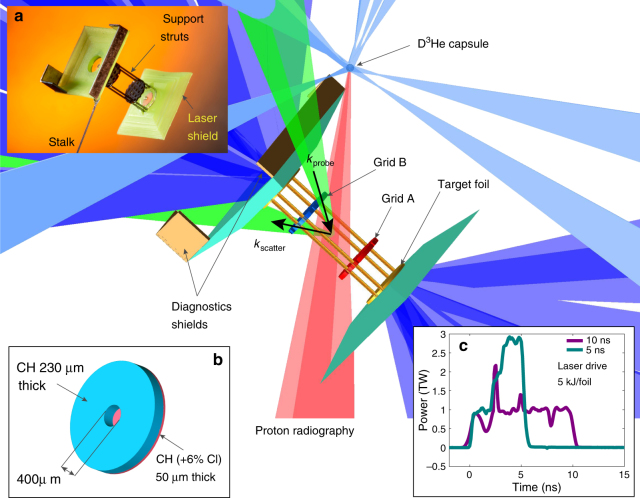
Experimental configuration. The main target (see photo in **a**) consists of two CH foils doped with 6% chlorine in atomic number (**b**) that are separated by 8 mm. Each foil is illuminated by ten 500 J, 1 ns pulse length, frequency tripled (351 nm wavelength) laser beams with 800 μm spot diameter. The beams are stacked in time to achieve the two pulse profiles shown in **c**. An additional set of 17 beams, all fired simultaneously, are used to implode a 420 μm diameter capsule consisting of a 2-μm-thick SiO_2_ shell filled with D_2_ gas at 6 atm and ^3^He at 12 atm. The implosion produces mono-energetic protons at 3.3 and 15 MeV with ~40 μm diameter source size, which traverse the plasma and are then collected by a CR-39 nuclear track detector with a total magnification factor of 28. The plasma expansion towards the center of the target is perturbed by the presence of two grids, placed 4 mm apart, with a 300 μm hole width and 300 μm hole spacing. Grid A has the central hole aligned on the center axis connecting the two foils, while grid B has the hole pattern shifted so that the central axis crosses the middle point between two holes. Thomson scattering uses a 30 J, 1 ns, frequency doubled (wavelength *λ* = 526.5 nm) laser beam to probe the plasma on the axis of the flow, 400 μm from the center and in a 50 μm focal spot, towards grid B. The scattered light is collected with 63° scattering angle and the geometry is such that the scattering wavenumber *k* = *k*_scatter_−*k*_probe_, where $\left| {k_{{\mathrm{scatter}}}} \right| \approx \left| {k_{{\mathrm{probe}}}} \right| = 2\pi {\mathrm{/}}\lambda$, is parallel to the axis of the flow

**Fig. 2:**
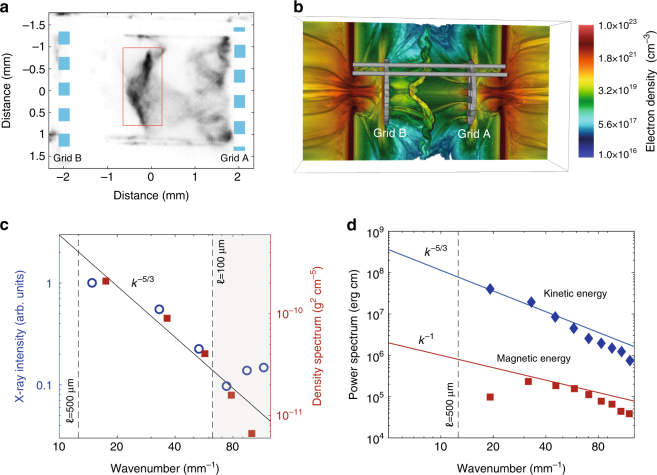
Characterization of the plasma turbulence. **a** X-ray pinhole image of the colliding flows at *t* = 35 ns after the laser drive, using the 5 ns pulse profile. The image was recorded onto a framing camera with ~1 ns gate width and filtered with 0.5 μm C_2_H_4_ and 0.15 μm Al. The pinhole diameter is 50 μm. **b** Rendering of the electron density from three-dimensional FLASH simulations at *t* = 35 ns. **c** The open blue circles give the power spectrum of the X-ray emission from the collision region, defined by the rectangular region shown in panel **a**. The power spectrum has been filtered to remove edge effects and image defects. Details of this procedure are given in Supplementary Methods. The shaded region at high wavenumbers is dominated by noise. The spectrum of the density fluctuations, as obtained from FLASH simulations in the turbulent region, is shown with red squares. **d** Blue diamonds: power spectrum of the kinetic energy from FLASH simulations. Red squares: power spectrum of magnetic energy from FLASH simulations. The simulated magnetic energy spectrum is considerably shallower than the Kolmogorov-like kinetic energy spectrum, as predicted by ref. ^23^ and other studies in the Pm < 1 regime (see text)

**Fig. 3:**
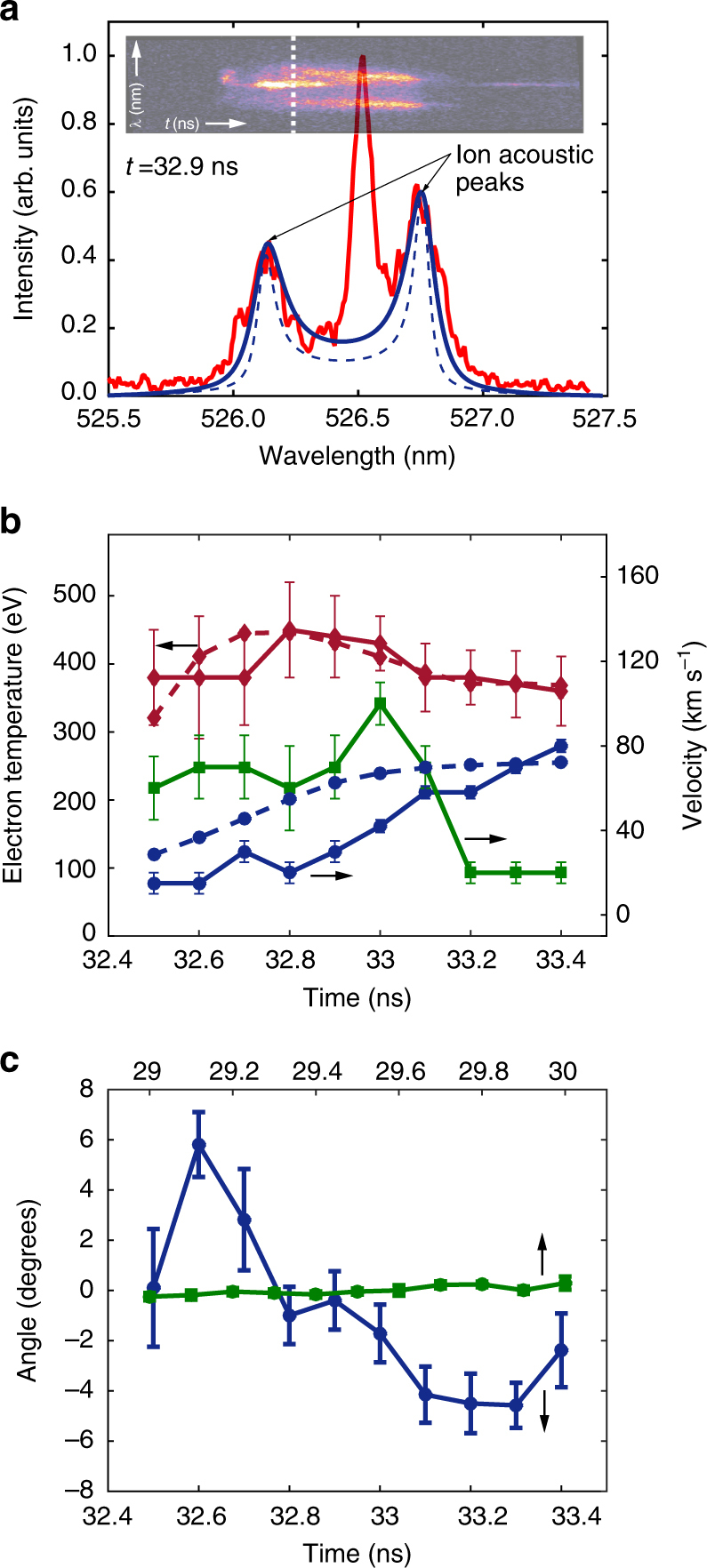
Thomson scattering measurements. Electron temperatures and flow velocities are obtained by fitting the experimental data with the frequency-dependent Thomson scattering cross-section^37^. In the fitting procedure we assumed an electron density of ≲10^20^ cm^−3^ (as predicted by FLASH simulations). At these electron densities, the frequency distribution of the scattered light does not depend on the electron density, which only provides an overall normalization factor. **a** Thomson scattering data (red solid line) at *t* = 32.9 ns obtained from a target driven with the 5 ns pulse profile. The blue dashed line corresponds to plasma in thermodynamic equilibrium (assuming equal electron and ion temperatures). The central peak is due to stray light at the probe laser wavelength (and it is used to determine the instrumental resolution of the spectrometer). The blue solid line corresponds to the case in which additional broadening due to turbulence is included in the fitting procedure. The inset in the top panel shows the time-streaked image of the Thomson scattered light. The resolution of the streak camera is ~50 ps and the Thomson scattering signal is fitted every 100 ps. **b** Flow velocity towards grid B (full blue circles), turbulent velocity (full green squares), and electron temperature (full red diamonds) as measured by Thomson scattering for the case of a target driven with the 5 ns laser profile. FLASH simulation results for the electron temperature and flow velocity in the probe volume are also reported in dashed lines. The error bars are estimated from the *χ*^2^ fit of the data. **c** Estimated Faraday rotation data from the Thomson scattering data. This was done by separating the scattered light into two orthogonal polarizations (see Supplementary Methods). The blue line corresponds to the same conditions as **b**, above. The green line was obtained from an experiment involving a single-flow, single-grid experiment only, when the magnetic field is expected to be significantly smaller (see Supplementary Figure 8 for the proton radiography results arising for a single-flow, single-grid experiment). The errors are determined by the standard deviation of the data within the shot

The experiments were performed at the Omega laser facility at the Laboratory for Laser Energetics of the University of Rochester^32^ using a combined platform that builds on our previous work on smaller laser facilities^15,30,31^. Laser ablation of a chlorine-doped plastic foil launches a plasma flow from its rear surface. The plasma then passes through a solid grid and collides with an opposite moving flow, produced in the same manner. In order to increase the destabilization of the motions as the flows collide, the two grids have hole patterns that are shifted with respect to each other. Further details on the experimental setup are given in Fig. 1. A set of diagnostics has been fielded to measure the properties of the flow, its turbulence and the magnetic field generated by it (see Figs. 2 and 3).

Extensive two-dimensional and three-dimensional simulations done prior to the experiments using the radiation-magnetohydrodynamics (MHD) code FLASH informed their design (see Fig. 2b and Supplementary Methods), including the details of the targets and the grids, and the timing of the diagnostics^33,34^.

### Characterization of the turbulent plasma

X-ray emission can be used to characterize the interaction of the colliding flows and assess properties of the resulting plasma inhomogeneities. The presence of a small amount of chlorine in the plasma enhances the emission in the soft wavelength region (<2 keV). Soft X-ray images taken at *t* = 35 ns from the start of the laser drive, which is after the flows collide, indicate a broad non-uniform spatial distribution of the emission over a region more than 1 mm across.

In order to characterize the state of the turbulent plasma produced by the collision of two laser-produced jets (as shown in Fig. 1), power spectra of the X-ray intensity fluctuations were extracted from the experimental data using a two-dimensional fast Fourier transform (see Fig. 2a, c). Under the assumption of isotropic statistics, fluctuations in the detected X-ray intensity are directly related to density fluctuations (see discussion in Supplementary Methods). The power spectrum of the density fluctuations extracted from the X-ray data is consistent with a Kolmogorov power law (*k*^−5/3^ scaling). Experimental data from other diagnostics indicate that the plasma motions are mainly subsonic (Mach number ≲1 at the outer scale); as a result, density fluctuations injected at large scales behave as a passive scalar and the spectra of the density and velocity fluctuations should be the same^35,36^. We conclude that the X-ray emission supports the notion that turbulent motions are present in the interaction region. This is also confirmed by FLASH simulations^34^, which predict subsonic motions of the plasma following the flow collision. Furthermore, the power spectrum of density and velocity fluctuations can be calculated directly from FLASH, and the results are consistent the same power law scaling for both (Fig. 2c, d).

The Thomson scattering diagnostic (see Fig. 1 and Supplementary Methods) allows us to measure simultaneously three different velocities associated with the flow^37^. First, the bulk plasma flow velocity—composed of a mean flow velocity *U* and outer-scale turbulent velocity *u*_*L*_—is obtained from the measurements of blueshifts (in frequency) of the scattered light resulting from the bulk plasma moving towards grid B. Second, the separation of the ion-acoustic waves is an accurate measure of the sound speed and thus of the electron temperature, *T*_e_. Third, the FLASH prediction of equal ion and electron temperatures allows us to infer from the broadening of the ion-acoustic features the turbulent velocity $u_\ell$ on the scale $\ell \sim 50$ μm (the Thomson scattering focal spot)^31,38^.

Based on these measurements, we find the following. Before the collision, the two plasma flows move towards each other with axial mean velocity $U \lesssim 200\, \mathrm {km}\, \mathrm {s}^{-1}$ in the laboratory rest frame, and have an electron temperature *T*_e_ ≈ 220 eV (see Supplementary Figure 1). After the collision, the axial flow slows down to 20–40 km s^−1^, with motions being converted into transverse components. The electron temperature increases considerably, reaching *T*_e_ ≈ 450 eV (Fig. 3). The measured time-averaged (RMS) turbulent velocity at scale $\ell$ is $u_\ell \sim 55\, \mathrm {km}\, \mathrm {s}^ {-1}$. If $u_\ell$ has Kolmogorov scaling, the turbulent velocity at the outer scale must therefore be $u_L \sim u_\ell (L{\mathrm{/}}\ell )^{1{\mathrm{/}}3} \approx 100\, \mathrm {km}\, \mathrm {s}^{-1}$. Electron density estimates can be obtained from the measured total intensity of the Thomson scattered radiation, to give a value *n*_e_ ≈ 10^20^ cm^−3^, which is also consistent with values predicted by FLASH simulations^34^. As shown in Supplementary Methods, plasmas with these parameters can be well described as being collisional, and in the resistive MHD regime.

For an MHD-type plasma, we can estimate the characteristic fluid and magnetic Reynolds numbers attained in our experiment. We find Re = *u*_*L*_*L*/*ν* ~ 1200 (*ν* is the viscosity), and Rm~600, using *L* ~ 600 μm, the characteristic driving scale determined by the average separation between grid openings. We have thus achieved conditions where Rm is comfortably larger than the expected critical magnetic Reynolds number required for turbulent dynamo^23^. The experiment also lies in the regime where the magnetic Prandtl number is Pm ≡ Rm/Re < 1.

### Magnetic field measurements

**Fig. 4:**
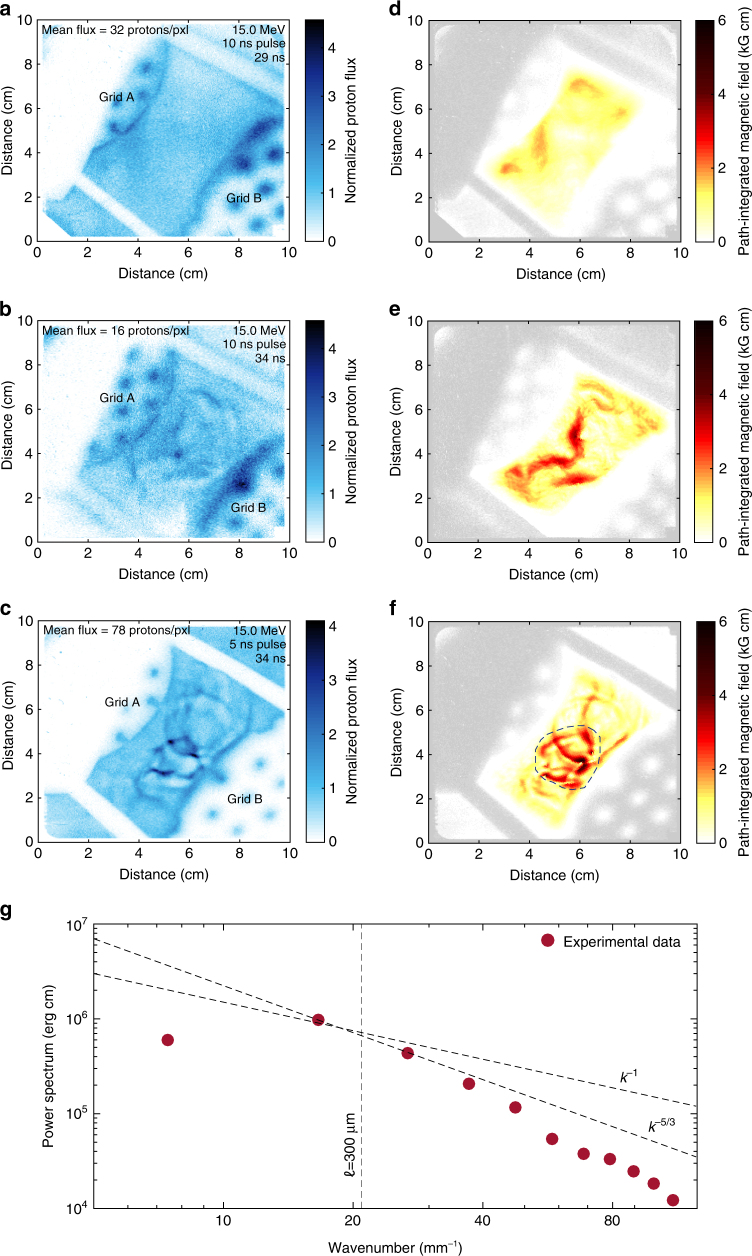
Proton radiography. **a** Normalized number of 15 MeV protons detected on a CR-39 plate. The normalization is such that unity corresponds to the mean number of protons per pixel on the detector. The D^3^He capsule was imploded at *t* = 29 ns. Fusion reactions occur 0.6 ns after the start of the implosion and the protons are emitted isotropically within a short burst, of ~150 ps duration^39^. The flight time of the protons to the plasma is 0.1 ns. The chlorinated plastic foils were driven with a 10 ns long pulse shape (see Fig. 1). X-ray data and FLASH simulations indicate that the plasma flows are close to collision by 29 ns (see also Supplementary Figure 9 in Supplementary Methods). Thus, this proton image can provide an estimate of the initial seed fields. **b** Same as **a**, but with the deuterium–tritium capsule imploded at *t* = 34 ns. The development of structures shows the development of fields in the interaction region. **c** Same as **b**, but with the chlorinated plastic foils driven with the 5 ns long pulse, which gives higher flow velocities, and hence higher magnetic Reynolds numbers. **d** Reconstruction of magnetic fields for case **a**. **e** Reconstruction of magnetic fields for case **b**. **f** Reconstruction of magnetic fields for case **c**. **g** Power spectrum of the magnetic energy from the reconstructed magnetic field from experimental data (the region bound by a dashed line in panel **f**)

Magnetic fields were inferred using both Faraday rotation (Fig. 3) and proton radiography (Fig. 4). The rotation of the polarization angle of Thomson scattered light provides a measure of the variation of the longitudinal component of the magnetic field integrated along the beam path, weighted by the electron density. Assuming a random field with correlation length $\ell _B$, we estimate *B*_||,rms_ ≈ 120(Δ*θ*/3^°^)(*n*_e_/10^20^ cm^−3^)^−1^$(\ell _n\ell _B{\mathrm{/}}0.2\,{\mathrm{m}{\rm m}}^2)^{ - 1{\mathrm{/}}2} \,\mathrm {kG}$, where *B*_||,rms_ is the root mean square (RMS) value of the magnetic field component parallel to the probe beam, Δ*θ* is the rotation angle, and $\ell _n\sim L \approx 0.6\,\mathrm {mm}$ is the scale length of the electron density along the line of sight. Estimating $\ell _B$ is more challenging, but as a reasonable estimate we can take the size of the grid aperture ($\ell _B \sim 300$ μm). The choice of $\ell _n\ell _B$ was corroborated by the FLASH simulations via synthetic Faraday rotation measurements (see Supplementary Figure 11 and the discussion in Supplementary Methods).

The appearance of strong, sharp features in proton radiographs provides independent evidence for large magnetic fields^39,40^. Such structures are the result of initially divergent proton rays produced by an imploding D^3^He capsule being focused by magnetic forces as they traverse the plasma. This leads, at the detector plane, to localized regions where the proton counts greatly exceed their average value and other regions where they are strongly depleted. The detailed spatial structure of the path-integrated magnetic field can be reconstructed from the experimental images assuming that the protons undergo small deflections as they pass through the plasma, and that the paths of neighboring protons do not cross before reaching the screen. Assuming isotropic statistics, this is sufficient information to calculate the power spectrum of the magnetic energy *E*_*B*_(*k*), and then the RMS magnetic field strength *B*_rms_ of fluctuating fields via $B_{{\mathrm{rms}}}^2 = 8\pi {\int} {\mathrm{d}}k\,E_B\left( k \right)$ (see refs. ^41,42^ and Supplementary Methods). Figure 4a shows that the magnetic field during the early phases of the collision is small, as no strong flux features appear in the radiographic image. The corresponding reconstructed RMS magnetic field strength *B*_rms_ obtained from Fig. 4d gives $B_{{\mathrm{rms}}} \lesssim 4\, {\rm kG}$.

## Discussion

Magnetic fields before the collision, and in the absence of any strong turbulence, are presumably Biermann battery fields produced at the laser spots and then advected by the flow, as indeed is confirmed by FLASH simulations. In contrast, Fig. 4b, c—corresponding to a later stage of the turbulent plasma’s lifetime for the 10 and 5 ns pulse shapes, respectively—do indeed show strong features, indicative of increased fields strengths and altered morphology. These strong features are absent in single-flow experiments (see Supplementary Figure 8 in Supplementary Methods), suggesting that the interaction of the counter-propagating flows and subsequent development of turbulence is essential for magnetic field amplification. The reconstruction algorithm can also be applied to the images in Fig. 4b, c (see Fig. 4e, f); in the latter case, we obtain *B*_rms_ ≈ 100 kG (see Supplementary Methods). This is consistent with our previous estimates based on Faraday rotation. We claim that this increase of the magnetic field during the collision cannot be simply explained by the compression of the field lines due to the formation of shocks (this would only account for a factor of two increase at most), nor by further generation by Biermann battery as the temperature gradients are not strong enough. This view is supported by FLASH simulations (see Supplementary Methods). Collisionless (magnetic field-generating) plasma processes such as the Weibel or filamentation instabilities cannot be responsible for the amplification of the magnetic field either, on account of the plasma’s high collisionality (see Supplementary Methods).

In Fig. 4g we show the spectrum of the magnetic energy, *E*_*B*_(*k*), calculated from the reconstructed path-integrated magnetic field. This is the spectrum on which the estimate of *B*_rms_ is based. The peak of this spectrum occurs at a wavenumber consistent with the claim that energetically dominant magnetic structures have a size $\ell _B \sim 300$ μm. The steeper slope of the spectrum at small wavelengths ($\lesssim 100$ μm) is not a property of the true spectrum, but is due to diffusion of the imaging beam caused by small-scale magnetic fields, and the underestimation of the magnetic energy by the reconstruction algorithm in the presence of small-scale caustics (see Supplementary Methods for a discussion of these effects). In the FLASH simulations the magnetic field spectrum appears to be consistent with a ~ *k*^−1^ power-law dependence, as shown in Fig. 2d, in agreement with the spectra of tangled fields near and above the dynamo threshold found in ref. ^23^ and by other investigators^43–45^, in the Pm < 1 regime.

Our experiment thus indicates that, as the two plasma flows collide, a strongly turbulent plasma, with magnetic Reynolds number above the threshold for dynamo action, is generated. The magnetic field grows from an initial value $B_{{\mathrm{rms}}} \lesssim 4$ to ~100–120 kG. We assume this to be near the saturated value because the Faraday rotation measurement begins over 2 ns (comparable to dynamical times) before the proton imaging diagnostic, and we infer similar magnetic field strengths from both. Note that the expected timescale for saturation to be reached is of the order of an outer-scale eddy-turnover time, *L*/*u*_*L*_ ~ 6 ns, a period that is comparable to the time that has elapsed between the initial flow collision and the magnetic field measurements. That the magnetized plasma is in a saturated state is corroborated by the FLASH simulation results (see Supplementary Figure 11 in Supplementary Methods).

If saturation is reached, the magnetic field energy should become comparable to the turbulent kinetic energy at the outer scale. We find $B_{{\mathrm{rms}}}^2{\mathrm{/}}\mu _0\rho u_L^2 \approx 0.04$ (where *ρ* is the plasma mass density and we have taken *B*_rms_ ≈ 120 kG). Because the field distribution is expected to be quite intermittent and because Rm in our experiment is unlikely to be asymptotically large compared to the dynamo threshold value, it is reasonable that the mean magnetic energy density is quantitatively smaller than the kinetic energy density^10,11,17^. However, a good indication that the magnetic field has reached a dynamically saturated state is that it is dynamically strong in the most intense structures, which are not necessarily volume filling. To find an upper experimental bound on the maximum field, *B*_max_, we assume that the deflections acquired by the imaging protons across the plasma come from an interaction with a single structure. The strongest individual structure in the reconstructed path-integrated image has scale $\ell _B\sim 140$ μm with a path-integrated field of 6 kG cm. This gives $B_{{\mathrm{max}}} \lesssim 430\,{\mathrm{kG}}$, which leads to $B_{{\mathrm{max}}}^2{\mathrm{/}}(\mu _0\rho u_L^2) \lesssim 0.5$, consistent with dynamical strength.

Our results appear to provide a consistent picture of magnetic field amplification by turbulent motions, in agreement with the longstanding theoretical expectation that turbulent dynamo is the dominant process in achieving dynamical equipartition between kinetic and magnetic energies in high magnetic Reynolds number plasmas found in many astrophysical environments.

## Methods

### Experimental facility and diagnostics

The laser-driven experiments presented in this work were carried out at the Omega laser facility at the Laboratory for Laser Energetics of the University of Rochester^32^, under the auspices of the National Laser User Facilities (NLUF) program of the U.S. Department of Energy (DOE) National Nuclear Security Administration (NNSA). In order to fully characterize the plasma state and measure the magnetic field amplification we fielded a number of experimental diagnostics, including Thomson scattering, X-ray imaging, Faraday rotation, and proton radiography. Detailed discussions on each diagnostic, along with full descriptions on how the experimental measurements were analyzed, are given in Supplementary Methods of the Supplementary Information.

### MHD and numerical simulations

The experimental platform was designed using radiation-MHD simulations with the publicly available, multi-physics code FLASH^46,47^. The numerical modeling of the platform employed the entire suite of High Energy Density Physics capabilities^33,34^ of the FLASH code. We performed an extensive series of moderate-fidelity 2D cylindrical FLASH radiation-MHD simulations on the Beagle 2 cluster at the University of Chicago followed by a smaller set of high-fidelity 3D FLASH radiation-MHD simulations on the Mira supercomputer at the Argonne National Laboratory. The simulation campaign is described in detail in a companion paper^34^ and presented in Supplementary Methods. The applicability of the MHD approximation and considerations regarding the collisionality of the turbulent plasma are discussed in Supplementary Methods of the Supplementary Information.

### Data availability

All data that support the findings of this study are available from the authors upon request.

## Electronic supplementary material

Supplementary Information
