# Supplementary material for: Laboratory evidence of dynamo amplification of magnetic fields in a turbulent plasma
Source: Nat Commun. 2018 Feb 9;9:591. doi: 10.1038/s41467-018-02953-2 (PMC5807305; doi:10.1038/s41467-018-02953-2)
Supplement: Supplementary file 1 — Supplementary Information [file 41467_2018_2953_MOESM1_ESM.pdf]

SUPPLEMENTARY INFORMATION

Laboratory evidence of dynamo amplification of magnetic fields in a turbulent plasma

Tzeferacos et al.

Supplementary Methods

Supplementary Method 1: Thomson Scattering

A Thomson scattering diagnostic was used to characterize various properties of the colliding jets. The Thomson scattering beam is a 30 J, 1 ns frequency doubled (526.5 nm wavelength) laser that probes the plasma within a 50 μm^2 region. The scattered light is collected with a 63° scattering angle and the geometry is such that the scattering wavenumber is parallel to the axis of the flow. When fitting the Thomson scattering data, a 0.025 nm instrument function was used and an electron density of $\lesssim$10^{20} cm^{-3} was assumed, as inferred from absolute calibration of the total scattered signal (see below) and predicted by FLASH simulations. Within a factor of 10 from this value for the electron density, the fitted Thomson scattering spectra showed no appreciable change. The resolution of the streak camera was $\sim$50 ps and so the Thomson scattering data was fitted every 100 ps.

We have investigated the potential plasma-heating effect resulting from the Thomson scattering probe beam. A simple estimate of this heating is given by [1]

$$\frac{\Delta T_{\rm e}}{T_{\rm e}} \lesssim 5.32 \times 10^{-7} \left(\frac{Z n_{\rm e}}{T_{\rm e}^{3/2}}\right) \lambda^3 \left(1 - {\rm e}^{-\hbar\omega/eT_{\rm e}}\right) I_0 \tau, \tag{1}$$

where $T_{\rm e}$ is the electron temperature (in eV), $n_{\rm e}$ the electron density (in cm^{-3}), Z the mean ion charge, λ the laser wavelength (in cm), ω the laser frequency, I_0 the probe laser intensity (in W cm^{-2}) and τ the laser pulse duration (in s). The above equation should be regarded as an upper bound, because it does not take into account the rapid transport of heat away from the laser focus by both convection and conduction. Indeed, the high electron thermal conductivity plays an important role in making the temperature uniform in the interaction region (see below). Taking an average value of $n_{\rm e} \sim 10^{20}$ cm^{-3} and assuming $T_{\rm e} \sim 450$ eV, we get $\Delta T_{\rm e}/T_{\rm e} \lesssim 20\%$. More quantitatively, the probe-heating effect was fully included in our FLASH simulations which predicted similar values to these simple estimates.

In order to observe the flow properties before jet collision, a single-jet experiment was carried out. The laser beams were fired on one side of the target only, and the Thomson scattering probe beam started 28 ns, after a 10 ns long laser drive. For this shot, the Thomson scattering

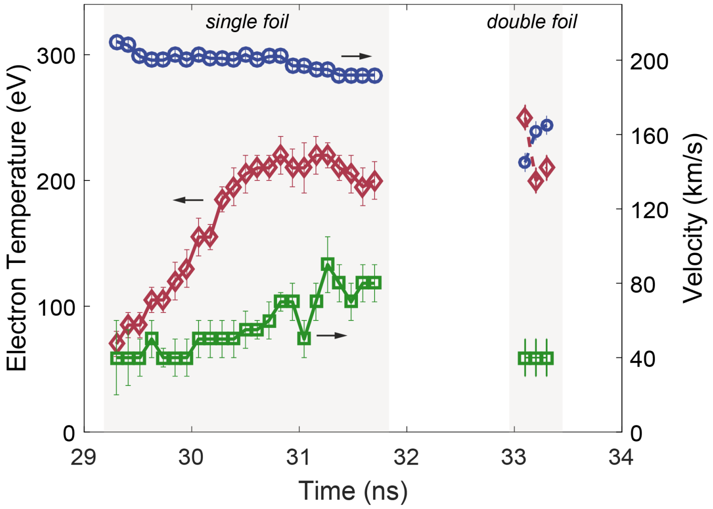

Supplementary Figure 1: **Thomson scattering from a 10 ns laser drive**. Flow velocity towards grid B (full blue circles), turbulent velocity (full green squares) and electron temperature (full red diamonds) as measured by Thomson scattering for the case of a 10 ns laser drive on both a single-jet and double-jet interaction. The error bars are determined in the same way as in Figure 3b of the main text.

laser beam probed the plasma on the axis of the flow, at the midpoint between the two targets. As shown in Supplementary Figure 1, before the collision, the jets had a velocity of $\sim$200 km s^{-1} and electron temperature $\sim$220 eV.

For direct comparison with the single-jet experiment, we also used the Thomson scattering diagnostic to determine flow properties from a double-grid shot, but with a 10 ns pulse shape. In this case, the probe beam was initiated with the same timing and positioning as was used with the 5 ns laser drive (described in the main text). The results are also shown in Supplementary Figure 1: the turbulent velocity for the 10 ns drive is lower than the 5 ns one, as is the associated electron temperature. Both are consistent with expected properties of the respective laser drives.

We have performed a full photometric calibration of the Thomson scattering system, recovering the electron density from each shot via a comparison between the incident and scattered power. Namely,

$$P_{\text{scatt}} = P_{\text{inc}}\, d\Omega\, r_0^2 \frac{\ell_{\text{TS}}}{2\pi} \left| \hat{\mathbf{s}} \times \left(\hat{\mathbf{s}} \times \hat{\mathbf{E}}_{\text{i0}} \right) \right|^2 S(k) Z^2 n_{\text{i}}, \tag{2}$$

where P_{scatt} is the scattered power, P_{inc} is the incident power of the probe beam, $d\Omega$ is the collecting solid angle, $r_0 = 2.818 \times 10^{-13}$ cm is the classical electron radius, $\ell_{\text{TS}} = 50\ \mu$m is the interaction length, $\hat{\mathbf{s}}$ is the unit Poynting vector, $\hat{\mathbf{E}}_{\text{i0}}$ is the probe beam's electric field unit vector, $S(k)$ is the spectral density function, Z is the mean ion charge and n_{i} is the ion density. The incident power is provided by the on-shot calorimetry performed at the Omega laser. The effective f-number of the optics was 9.1, giving a solid angle of 10^{-2} sr. The spectral density

function is [1]

$$S(k) = \frac{Z\alpha^4}{(1+\alpha^2)\left[1+\alpha^2\left(1+Z\right)\right]}, \tag{3}$$

which can be calculated using the previous Thomson scattering fits to the data. Here α is $1/k\lambda_{\mathrm{D}}$, with λ_{D} the Debye length. In our experiment we have $\alpha \gg 1$; in this limit Landau damping vanishes [1]. We can therefore interpret the Thomson scattering signal using the Salpeter approximation. The shape of the ion feature (i.e., the low-frequency part of the scattering spectrum due to the ion motions) is then a function of the parameter $\beta^2 = Z\left(\frac{\alpha^2}{1+\alpha^2}\frac{T_{\mathrm{e}}}{T_{\mathrm{i}}}\right) \sim Z$ [1]. For carbon ions and nearly equal electron and ion temperatures, $\beta \sim 2.4 < 3.45$ (see also p. 223 in Ref. 37). In this case, the real part of dielectric function has no real zeros and the spectrum consists of a wide (Doppler broadened) central feature and two peaks at the wavelengths corresponding to the minima in the dielectric function. The scattered power was determined via full photometric calibration of all the optics to convert the number of detected counts (on the CCD camera) to energy. We find that for the collided jets with a 5 ns drive, the electron density was $n_{\mathrm{e}} = Zn_{\mathrm{i}} \approx 7\times10^{19}\ \mathrm{cm}^{-3}$, in agreement with FLASH predictions (see Supplementary Figure 2).

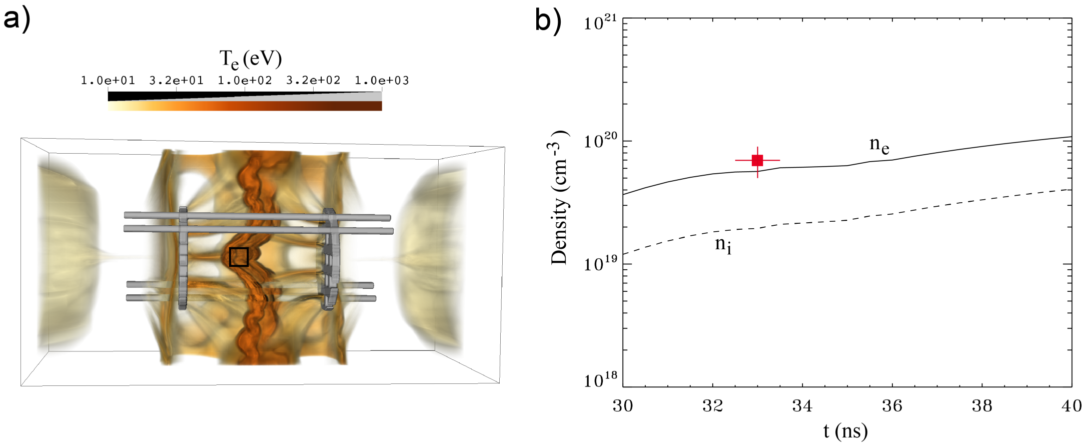

Supplementary Figure 2: **FLASH temperature and density variations in the plasma**. (a) Volume rendering of T_{e} for the 5 ns laser drive at $t = 35$ ns. (b) Electron density (solid line) and ion density (dashed line) profiles averaged in the jet interaction volume [the square box in panel (a)]. The red square corresponds to the estimated electron density from Thomson scattering photometric calibration. The vertical error is determined by the standard deviation from different calibration shots. The horizontal error bar is determined by the fact the probe laser has a 1 ns pulse length.

Supplementary Method 2: X-ray Imaging and Power Spectrum of Density Fluctuations

The effects of turbulence on the X-ray emissivity were investigated using images of the plasma obtained from a framing camera. These images have a pixel size of $\delta x \approx 9\,\mu\mathrm{m}$, with an estimated resolution $l \approx 50 - 100\,\mu\mathrm{m}$ from the pinhole size and detector sensitivity. We characterized relative fluctuations in detected intensity by performing spectral analysis of the jet-interaction region, visible approximately half-way between the grids (see Figure 2 of the main text). In order to distinguish small-scale density variation from large-scale inhomogeneities of the interaction region, we constructed relative-intensity maps based on 'coarse-grained' mean fields calculated via a 61×61 pixel smoothing filter – corresponding to an outer length scale $L \approx 600\,\mu\mathrm{m}$ – combined with a two-dimensional window function to remove edge effects. Power spectra were then calculated by taking average values from a binned annular histogram of a two-dimensional fast Fourier transform (FFT) applied to the relative-intensity image. To reduce the effect of spectral distortion due to image defects [2] such as neutron hits or striations, various filtering methods were employed. Extreme outliers were systematically detected by testing deviation from surrounding points, then removed using a median filter. Localized defects tend to appear as anisotropic features in Fourier space. Their impact on the assigned power-spectrum values was mitigated by using the median, rather than the mean, as a measure of central tendency for each bin: the former is more robust against the outlying values typically associated with such features. The efficacy of these methods was successfully tested by applying such defects on simulated Gaussian fields with prescribed spectra. To check the consistency of the spectra obtained, the above analysis was performed on multiple regions in the image, and various mean-field estimation methods were employed, including a variety of windowed regions. The jump observed in the experimental spectrum at high wavenumbers (Figure 2c of the main text) is most likely due to Poisson noise. This follows because, in the two-dimensional spectrum, Poisson noise manifests itself as a constant feature: the distortion to the predicted 1D spectrum is proportional to the wavenumber.

Fluctuations in the detected X-ray intensity are typically related to density fluctuations [3], which in turn can be related to velocity perturbations under certain conditions. The radiative energy flux (emissivity) from an optically thin plasma with $T = T_\mathrm{e} = T_\mathrm{i}$ is given by a cooling function [4]

$$\epsilon = \rho \kappa_\mathrm{P} \sigma T^4 = \kappa_0 \sigma \rho^{\alpha+1} T^{\beta+4}, \tag{4}$$

where σ is the Stefan-Boltzmann constant, ρ the mass density, and the functional form of the Planck opacity has been assumed to be $\kappa_\mathrm{P} = \kappa_0 \rho^\alpha T^\beta$, with κ_0 a suitable constant. For Bremsstrahlung-dominated radiation, $\beta = -7/2$ and $\alpha = 1$, resulting in weak dependence on temperature [4]. Since $\rho \propto n$, we conclude that the emissivity can be written as

$$\epsilon = C n^{\alpha+1} T^{\beta+4}, \tag{5}$$

where C is a constant. Decomposing the number density and temperature into mean and fluc-

tuating parts, viz.,

$$n = n_0 + \delta n, \quad T = T_0 + \delta T, \tag{6}$$

and assuming $n_0 \gg \delta n,\ T_0 \gg \delta T$, we find that the emissivity becomes

$$\begin{aligned} \epsilon = \epsilon_0 &\left[1 + (\alpha+1)\,\frac{\delta n}{n_0} + (\beta+4)\,\frac{\delta T}{T_0}\right] \\ &+ \epsilon_0\left[\frac{(\alpha+1)\,(\alpha+2)}{2}\frac{\delta n^2}{n_0^2} + \frac{(\beta+4)\,(\beta+5)}{2}\frac{\delta T^2}{T_0^2}\right] + \mathcal{O}\left(\frac{\delta n^3}{n_0^3}, \frac{\delta T^3}{T_0^3}\right), \end{aligned} \tag{7}$$

where the mean emissivity is $\epsilon_0 = C n_0^{\alpha+1} T_0^{\beta+4}$. The intensity detected on the screen is then given by the line-of-sight integral

$$\begin{aligned} I &= \int_0^{\ell_n} \epsilon(x,y,z)\ \mathrm{d}z \\ &\approx I_0\ +\ (\alpha+1)\int_0^{\ell_n} \epsilon_0(x,y,z)\,\frac{\delta n}{n_0}\,\mathrm{d}z\ +\ (\beta+4)\int_0^{\ell_n} \epsilon_0(x,y,z)\,\frac{\delta T}{T_0}\,\mathrm{d}z, \\ &+\ \frac{(\alpha+1)\,(\alpha+2)}{2}\int_0^{\ell_n} \epsilon_0(x,y,z)\,\frac{\delta n^2}{n_0^2}\,\mathrm{d}z\ +\ \frac{(\beta+4)\,(\beta+5)}{2}\int_0^{\ell_n} \epsilon_0(x,y,z)\,\frac{\delta T^2}{T_0^2}\,\mathrm{d}z, \end{aligned} \tag{8}$$

with mean-field intensity I_0 defined by $I_0 \equiv \int_0^{\ell_n} \epsilon_0(x,y,z)\,\mathrm{d}z$, where ℓ_n is the size of the plasma. Assuming ℓ_n to be larger than the scale of the dominant fluctuations l, and homogeneity at small scales (an assumption discussed at the end of this subsection), we can separate the various terms with a WKB-type (Wentzel - Kramers -Brillouin) approximation to give

$$\begin{aligned} \frac{\delta I}{I_0}(x,y) \approx \frac{(\alpha+1)}{\ell_n}\int_0^{\ell_n} \frac{\delta n}{n_0}\,\mathrm{d}z\ +\ \frac{(\beta+4)}{\ell_n}\int_0^{\ell_n} \frac{\delta T}{T_0}\,\mathrm{d}z \\ +\ \frac{(\alpha+1)\,(\alpha+2)}{2\ell_n}\int_0^{\ell_n} \frac{\delta n^2}{n_0^2}\,\mathrm{d}z\ +\ \frac{(\beta+4)\,(\beta+5)}{2\ell_n}\int_0^{\ell_n} \frac{\delta T^2}{T_0^2}\,\mathrm{d}z. \end{aligned} \tag{9}$$

Note that we cannot necessarily neglect the quadratic terms in Supplementary Equation 9, because the linear integrals are reduced in magnitude by a factor $(l/\ell_n)^{1/2}$ due to the fluctuating integrands; this reduction is not seen in the quadratic terms. In order to ignore the latter, we would require $\delta n/n_0 < (l/\ell_n)^{1/2}$ and $\delta T/T_0 < (l/\ell_n)^{1/2}$. Yet, for 1D power spectra shallower than k^{-2} this condition is not satisfied.

To proceed further, we need to use an equation of state to relate density and temperature fluctuations. For example, in the case of subsonic, ideal gas motions, it can be shown from pressure balance that

$$\frac{\delta n}{n_0} \approx -\frac{\delta T}{T_0}. \tag{10}$$

This gives a quadratic relation between intensity and density fluctuations of the form

$$\frac{\delta I}{I_0}(x,y) \approx \frac{c_1}{\ell_n}\int_0^{\ell_n} \frac{\delta n}{n_0}(x,y,z)\ \mathrm{d}z + \frac{c_2}{\ell_n}\int_0^{\ell_n} \frac{\delta n^2}{n_0^2}(x,y,z)\ \mathrm{d}z, \tag{11}$$

provided c_1 and c_2 do not vanish. Alternatively, a similar result can be obtained by assuming that the temperature remains uniform in the plasma, thus neglecting temperature fluctuations with respect to density fluctuations. Since thermal conductivity is large in our experiment, such an assumption would be reasonable; furthermore, a relatively uniform temperature across the interaction region is predicted by FLASH simulations (see Supplementary Figure 2). The above considerations allow the (assumed homogeneous) intensity and density correlation functions, defined by

$$C^{(I)}\left(\mathbf{x}_\perp' - \mathbf{x}_\perp\right) = \left\langle \frac{\delta I}{I_0}(\mathbf{x}_\perp)\, \frac{\delta I}{I_0}(\mathbf{x}_\perp') \right\rangle , \tag{12}$$

$$C^{(n)}\left(\mathbf{x}' - \mathbf{x}\right) = \left\langle \frac{\delta n}{n_0}(\mathbf{x})\, \frac{\delta n}{n_0}(\mathbf{x}') \right\rangle , \tag{13}$$

$$C^{\left(n^2\right)}\left(\mathbf{x}' - \mathbf{x}\right) = \left\langle \frac{\delta n^2}{n_0^2}(\mathbf{x})\, \frac{\delta n^2}{n_0^2}(\mathbf{x}') \right\rangle , \tag{14}$$

to be related via

$$C^{(I)}\left(\mathbf{x}_\perp' - \mathbf{x}_\perp\right) = \frac{c_1^2}{\ell_n}\int_{-\infty}^{\infty} C^{(n)}\left(\mathbf{x}'_\perp - \mathbf{x}_\perp, r_z\right)\, \mathrm{d}r_z, + \frac{c_2^2}{\ell_n}\int_{-\infty}^{\infty} C^{\left(n^2\right)}\left(\mathbf{x}'_\perp - \mathbf{x}_\perp, r_z\right)\, \mathrm{d}r_z, \tag{15}$$

where we have used typical decay conditions on the density correlation function to extend the integrals formally over the entire real line, and have neglected the cross-correlation between $\delta n/n_0$ and $\delta n^2/n_0^2$ on the grounds that such a term contains the same reduction factor as the linear term, and so is negligible under the small fluctuation assumption $\delta n/n_0 \ll 1$.

The 2D intensity spectrum $P_{\mathrm{2D}}(\mathbf{k}_\perp)$, 3D density spectrum $P_{\mathrm{3D}}(\mathbf{k})$, and 3D squared-density spectrum $P_{\mathrm{3D}}^{(2)}(\mathbf{k})$, given by the Fourier transforms of Supplementary Equations 12, 13, and 14, respectively, combined with the Wiener-Khinchin Theorem [5], are therefore related by

$$P_{\mathrm{2D}}(\mathbf{k}_\perp) = \frac{2\pi c_1^2}{\ell_n} P_{\mathrm{3D}}(\mathbf{k}_\perp, 0) + \frac{2\pi c_2^2}{\ell_n} P_{\mathrm{3D}}^{(2)}(\mathbf{k}_\perp, 0) = \frac{2\pi c_1^2}{\ell_n} P_{\mathrm{3D}}(k_\perp) + \frac{2\pi c_2^2}{\ell_n} P_{\mathrm{3D}}^{(2)}(k_\perp) \, . \tag{16}$$

Isotropy of the statistics of the power spectrum was invoked for the last equality, and is justified subsequently. We simplify Supplementary Equation 16 by noting that $P_{\mathrm{3D}}^{(2)}(k)$ can be written

$$P_{\mathrm{3D}}^{(2)}(k) \equiv \left\langle \left| \left(\frac{\delta n^2}{n_0^2}\right)_{\mathbf{k}} \right|^2 \right\rangle = \left\langle \frac{1}{n_0^4} \left| \sum_{\mathbf{k}'} \delta n_{\mathbf{k}'}\, \delta n_{\mathbf{k}-\mathbf{k}'} \right|^2 \right\rangle = \frac{1}{n_0^4} \sum_{\mathbf{k}',\mathbf{k}''} \left\langle \delta n_{\mathbf{k}'}\, \delta n_{\mathbf{k}-\mathbf{k}'}\, \delta n^*_{\mathbf{k}''}\, \delta n^*_{\mathbf{k}-\mathbf{k}''} \right\rangle \, . \tag{17}$$

On account of the largest density fluctuations occurring at the smallest wavenumbers, the sum on the RHS of Supplementary Equation 17 is dominated by wavemodes where $k', k'' \ll k$. This is equivalent to assuming that the 1D power spectrum is decreasing, so that $|\delta n_{\mathbf{k}_1}| > |\delta n_{\mathbf{k}_2}|$ if $|\mathbf{k}_1| < |\mathbf{k}_2|$; and as presented in the main text, for $k \gg 2\pi/\ell_n$ the 1D power spectrum is indeed

decreasing. Under this assumption, Supplementary Equation 17 becomes

$$P_{\rm 3D}^{(2)}(k) \approx \frac{1}{n_0^4} \sum_{\mathbf{k}',\mathbf{k}''} \langle \delta n_{\mathbf{k}'}\, \delta n_{\mathbf{k}}\, \delta n^*_{\mathbf{k}''}\, \delta n^*_{\mathbf{k}} \rangle \approx \left\langle \left| \frac{\delta n_{\mathbf{k}}}{n_0} \right|^2 \right\rangle \sum_{\mathbf{k}',\mathbf{k}''} \left\langle \frac{\delta n_{\mathbf{k}'}}{n_0} \frac{\delta n^*_{\mathbf{k}''}}{n_0} \right\rangle = P_{\rm 3D}(k) \left\langle \frac{\delta n^2}{n_0^2} \right\rangle . \tag{18}$$

In short, for a decaying spectrum with small density fluctuations at $k \gg 2\pi/\ell_n$ the contribution of $P_{\rm 3D}^{(2)}(k)$ to $P_{\rm 2D}(k)$ is simply to modify its amplitude. The desired relation

$$P_{\rm 3D}(k) \propto P_{\rm 2D}(k) \tag{19}$$

follows from Supplementary Equation 18 applied to Supplementary Equation 16.

In the subsonic regime, this relationship should hold more generally than for an ideal gas, provided any linearized relationship deduced between density and temperature fluctuations from the equation of state does not lead to the coefficients c_1 and c_2 vanishing in the quadratic-intensity result, Supplementary Equation 11.

The approach described here was validated against numerical results. We have created a synthetic X-ray emission image (Supplementary Figure 3a) using FLASH simulation results, post-processed with the multi-dimensional collisional-radiative spectral analysis code SPECT3D (http://www.prism-cs.com). Supplementary Equation 18 can be tested with simulated stochastic density fields, and numerical results support it. For example, Supplementary Figure 3c shows a comparison of $P_{\rm 3D}(k)$ to $P_{\rm 3D}^{(2)}(k)$ for the density field resulting from FLASH simulations. At large wavenumbers, the slopes of the density and squared-density spectra are similar, in line with expectations from Supplementary Equation 18. We note that at such wavenumbers, $P_{\rm 3D}^{(2)}(k)$ is greater in magnitude than $P_{\rm 3D}(k)$, confirming the importance of keeping quadratic terms in the expansion in Supplementary Equation 9. The discrepancy at smaller wavenumbers is likely indicative of the breakdown of the decreasing-spectrum approximation inherent in Supplementary Equation 18.

Supplementary Figure 3b also provides a numerical test of Supplementary Equation 16, by comparing for FLASH simulations the predicted 2D relative intensity spectrum to the true intensity spectrum, assuming radiation is Bremsstrahlung dominated. With the exception of the lowest wavenumbers, reasonable agreement is again seen. The difference between the two spectra is presumed to arise from the fact that in the FLASH simulations the size of relative density and intensity fluctuations on the largest scales are not asymptotically small: $(\delta n/n_0)_{\rm rms} \approx 0.5$, and $(\delta I/I_0)_{\rm rms} \approx 0.3$. We believe that the disagreement for small wavenumbers is not a source of concern, on the grounds that the calculated spectrum at such scales is already somewhat uncertain due to $k\ell_n \sim 1$. To summarize, the numerical tests using FLASH simulations show that inferring the true density spectrum from the experimental intensity spectrum using Supplementary Equation 16 seems to be a sensible approach.

In deriving Supplementary Equation 19, we assumed that the quantities $\delta n/n_0$ and $\delta I/I_0$ possess homogeneous and isotropic statistics. These are non-trivial assumptions, as the experiment does not produce an infinite plasma; and while the target is close to being symmetric

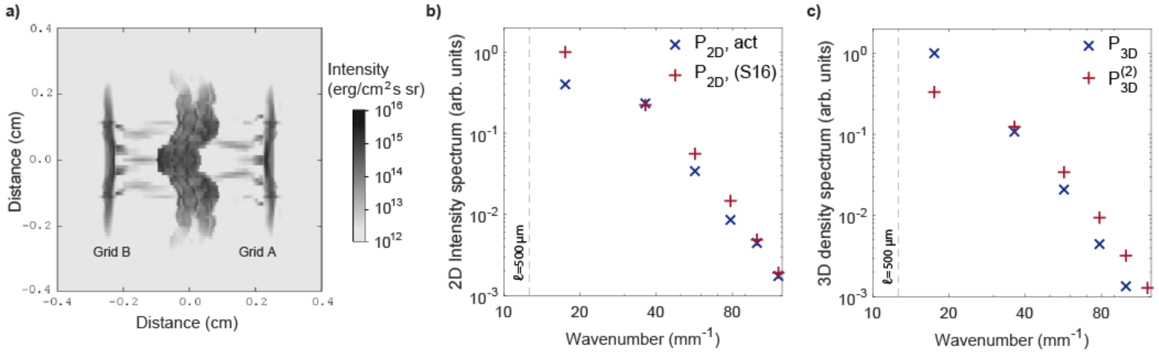

Supplementary Figure 3: **Testing intensity and density spectral relations in FLASH**. a) Synthetic x-ray emission image for the 5 ns laser drive at $t = 35$ ns. The image has been generated with the code SPECT3D using FLASH simulation results. b) Comparison of the exact intensity spectrum resulting from FLASH simulations in the case of Bremsstrahlung-dominated radiation ($\beta = -7/2$ and $\alpha = 1$) to the intensity spectrum predicted by Supplementary Equation 16. c) Comparison of the 3D density spectrum to the 3D squared-density spectrum, as calculated from FLASH simulations. According to Supplementary Equation 18, at sufficiently large wavenumbers these spectra should be proportional to each other. The region from the FLASH simulations used to calculate these spectra is the same as the region used to calculate the 1D density spectrum shown in Figure 2c of the main text.

around the direction parallel to the initial jet flow direction (hereon referred to as the line of centers), the directions parallel and perpendicular to the line of centers could potentially lead to anisotropic plasma statistics. However, these assumptions can be tested using the experimental images, whose horizontal axis is parallel to the line of centers, and vertical axis perpendicular. First, if emission statistics were inhomogeneous, we would expect to obtain different spectral slopes from neighboring regions. Supplementary Figure 4a shows this not to be the case, with close agreement in the spectra calculated from three adjacent regions composing the rectangular region denoted in Figure 2b of the main text. Second, for anisotropic intensity statistics, the spectral slopes obtained by averaging sectors in k-space predominantly parallel and perpendicular to the line of centers respectively would be distinct. Supplementary Figure 4b refutes this by showing that the density spectra calculated using such a procedure have the same slope.

Supplementary Method 3: Faraday Rotation

We have implemented Faraday rotation measurements in the plasma using the Thomson scattering diagnostic system already available at the Omega laser facility. Using a Wollaston prism, the Thomson scattered light beam was separated into two orthogonal components, S and P. The magnitude of the detected S and P polarizations I_{S} and I_{P} depends on three factors: the initial polarization of the linearly-polarized probe beam, the relative transmission of the S and P com-

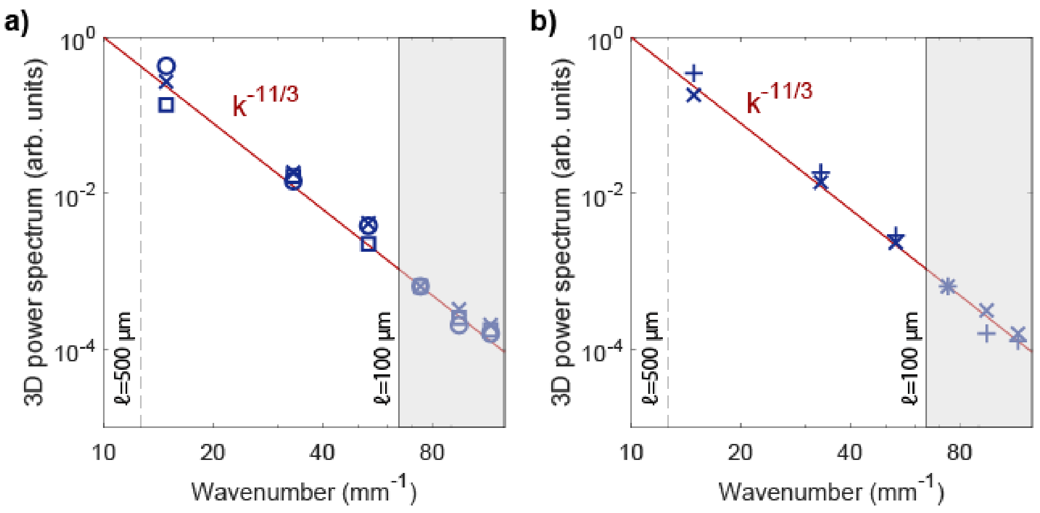

Supplementary Figure 4: **Testing homogeneity and isotropy assumptions for density spectrum**. (a) Test of homogeneity assumption for the density spectrum, performed by dividing the region of the X-ray image selected for analysis (denoted in Figure 2b of the main text by the red rectangle) into three equally sized regions, then calculating the predicted 3D density spectra for each. Blue saltires denote the upper region, squares the middle region, and circles the lower region. For comparison, the spectra are plotted with a 3D Kolmogorov power law. The upper wavenumber cutoff denotes the nominal resolution limit of the X-ray imaging diagnostic. (b) Test of isotropy assumption for the density spectrum. The test consists of calculating the two-dimensional FFT applied to the full region denoted in Figure 2b of the main text, but calculating partial rather than full binned annular histograms from the FFT. Wavenumbers are separated into those whose predominant direction is horizontal, and vertical, corresponding to directions parallel and perpendicular to the initial direction of the plasma flows (the line of centers). The parallel and perpendicular spectra (denoted by crosses and saltires respectively) are then calculated using the average values from each of the partial annular regions.

ponents through the diagnostic optics subsequent to their separation by the prism, and changes to polarization due to Faraday rotation. Namely,

$$I_{\mathrm{S}} = A_{\mathrm{S}}\, I_0 \sin^2\left(\theta_{\mathrm{c}} + \Delta\theta\right), \tag{20}$$

$$I_{\mathrm{P}} = A_{\mathrm{P}}\, I_0 \cos^2\left(\theta_{\mathrm{c}} + \Delta\theta\right), \tag{21}$$

where I_0 is the probe laser intensity, A_{S} and A_{P} the (distinct) transmission factors for each polarization, θ_{c} the angle at which the S and P components are split by the prism, and $\Delta\theta$ the rotation due to the Faraday effect. Taking the ratio of Supplementary Equations 20 and 21, we find

$$\tan^2\left(\theta_{\mathrm{c}} + \Delta\theta\right) = \frac{A_{\mathrm{P}}}{A_{\mathrm{S}}}\frac{I_{\mathrm{S}}}{I_{\mathrm{P}}}. \tag{22}$$

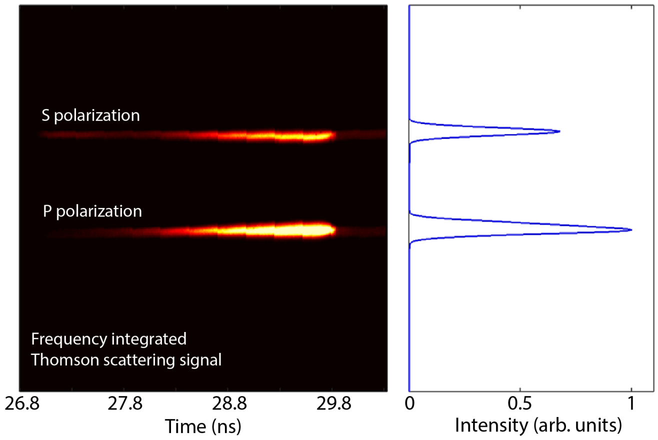

Supplementary Figure 5: **Faraday-rotation calibration**. Total Faraday-rotation signal for the two polarizations for the case of a single jet driven with a 10 ns pulse profile.

If θ_c and the ratio A_S/A_P are known, the degree of Faraday rotation $\Delta\theta$ can be calculated from I_S/I_P.

In this experiment, the Faraday rotation diagnostic was set up so that the S and P components are equally split by the prism, that is $\theta_c = 45^o$. To calculate the transmission ratio, we consider the magnitude of the two components for a single jet case. The single-jet case can be used for calibration, because the proton radiography diagnostic indicates that the magnetic fields are small enough that the Faraday rotation angle is below detector sensitivity. In Supplementary Figure 5, we show the raw image of the split polarizations and plot the summed intensity of the signal for its entire duration. We determine the transmission ratio to be $A_S/A_P = 0.43$. The use of this data for calibration is validated by the fact that the measured rotation angle remains constant in time, as shown in Figure 3c of the main text. Since the resolution of the streak camera is ~50 ps, the Faraday rotation angle for other shots is then calculated at 100 ps intervals.

When the Faraday-rotation angle is small, it is given (in Gaussian units) by [5]

$$\Delta\theta = \frac{\lambda^2 e^3}{2\pi m_e^2 c^4} \int_0^{2\ell_n} n_e(s)\, B_\parallel(s)\, \mathrm{d}s. \tag{23}$$

It is appropriate to use twice the size of the plasma ℓ_n for the path length of the integral because the geometry of the jet-interaction region is such that the Thomson scattering volume lies on the opposing side to that from which the probe beam originates. The incident laser's wavelength is $\lambda = 5.27 \times 10^{-5}$ cm, so this implies

$$\int_0^{2\ell_n} n_e(s)\, B_\parallel(s)\, \mathrm{d}s = 2.4 \times 10^{23} \Delta\theta \text{ G cm}^{-2}. \tag{24}$$

If the mean magnetic field is small or zero, the mean of the Faraday-rotation measure should vanish and so measured values of it correspond to the standard deviation of the line-of-sight integral in Supplementary Equation 24. This can be estimated by a random-walk argument: assuming a random field has correlation scale ℓ_B, the typical deviation is equal to the deviation acquired across one correlated structure multiplied by the square root of the number of such structures encountered. Supplementary Equation 24 then implies

$$\sqrt{\ell_n \ell_B}\, n_{\mathrm{e}}\, B_{\|} \sim 1.7 \times 10^{23} \Delta\theta \ \mathrm{G\,cm^{-2}}, \tag{25}$$

where ℓ_n is the length scale of the density profile and ℓ_B the scale of the magnetic structures that have the largest amplitude. This estimate can be re-arranged to give the result stated in the main text.

There are a range of effects to which the probe beam is subject but that can be reasonably neglected in our analysis. The probe laser changes frequency – and hence wavelength – after scattering, due to collective plasma effects in the probe-interaction region; however, the largest such change will be of the order of the plasma frequency, and this corresponds to a maximum wavelength shift $\Delta\lambda/\lambda \lesssim 30\%$. The error in Faraday rotation associated to this wavelength modification is $\lesssim 20\%$, which is less than the noise in the streak camera data. Due to the small size of the electron Larmor frequency ($\Omega_{\mathrm{e}} \approx 2 \times 10^{12}\,\mathrm{s^{-1}}$) compared to the laser frequency ($\omega \approx 4 \times 10^{15}\,\mathrm{s^{-1}}$), it can also be shown that other magnetic effects on the polarization – such as the Cotton-Mouton effect – are much smaller in magnitude than the experimental error [6] and can be safely omitted.

Supplementary Method 4: Proton Radiography

Proton deflections in the proton radiography diagnostic are primarily due to magnetic fields rather than electric fields or effects such as Coulomb collisions and kinetic beam instabilities. This can be seen from the energy map on the CR-39 plates. Supplementary Figure 6 shows that, in the jet-interaction region, the mean proton energy has a uniform distribution without pronounced density structures.

For arbitrary magnetic-field configurations and imaging beam parameters, relating analytical distributions of flux and the magnetic fields from which they arise is non-trivial. However, for radiographic setups similar to (and including) this experiment, a number of simplifications can be made to improve tractability of the "reconstruction" problem of extracting path-integrated magnetic fields directly from a given proton flux distribution. Based on such an approach, here we discuss the formulas used to derive the images of path-integrated fields, shown in Figure 4 of the main text, as well as the assumptions under which these formulas are valid. A full derivation of these results is presented elsewhere [7].

We calculate path-integrated fields from the experimentally obtained proton-flux images, assuming that the distribution of flux Ψ is related to the path-integrated field by a Monge-

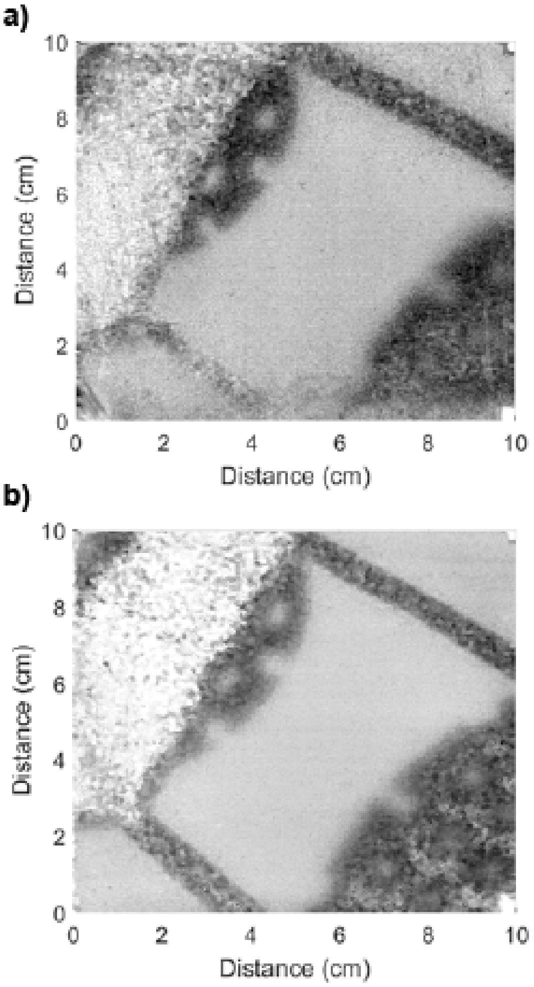

Supplementary Figure 6: **Mean proton energy**. Mean proton energy *vs.* position for the 15.0 MeV protons recorded on the CR-39 plate. The distribution is very uniform, indicating that the structures seen in the flux images (see Figures 4c and 4d of the main text) are due to deflections of protons by magnetic fields. a) Target with both grids, and with the two chlorinated plastic foils driven with a 10 ns long pulse shape. The D^3He capsule was imploded at $t = 34$ ns. b) same as a) but with a 5 ns laser driver

Ampère equation [8] of the form

$$\Psi(\nabla\phi(\mathbf{x}_{\perp 0})) = \frac{\Psi_0}{|\det \nabla\nabla\phi(\mathbf{x}_{\perp 0})|}\,. \tag{26}$$

In this expression, $\nabla \equiv \partial/\partial\mathbf{x}_{\perp 0}$ is a gradient operator defined on a two-dimensional coordinate system $\mathbf{x}_{\perp 0}$ co-planar with the imaging screen (CR-39 plate), but with a scale reduced by the

imaging magnification factor

$$\mathcal{M} \approx \frac{r_\mathrm{s}+r_\mathrm{i}}{r_\mathrm{i}} \approx 28\,, \tag{27}$$

where $r_\mathrm{s} \approx 27\,\mathrm{cm}$ is the distance from the plasma to the screen and $r_\mathrm{i} \approx 1\,\mathrm{cm}$ is the distance from the proton source to the plasma. Ψ_0 is the initial uniform flux distribution, but with its magnitude multiplied by a scaling factor of $\mathcal{M}^{-2}$. $\nabla\phi$ is a two-dimensional gradient map related to the path-integrated magnetic field by

$$\nabla\phi(\mathbf{x}_{\perp 0}) = \mathbf{x}_{\perp 0} + \frac{r_\mathrm{s} r_\mathrm{i}}{r_\mathrm{s}+r_\mathrm{i}} \frac{e}{m_\mathrm{p} c v_\mathrm{p}} \hat{\mathbf{z}} \times \int_0^{l_\mathrm{i}} \mathrm{d}s\, \mathbf{B}[\mathbf{x}(s)]\ , \tag{28}$$

where e is the proton charge, m_p the proton mass, v_p the proton speed, $\hat{\mathbf{z}}$ a unit vector perpendicular to the imaging screen, l_i the size of the plasma, s the path length, and $\mathbf{x}(s)$ the trajectory of a proton through the plasma with initial position $\mathbf{x}_0 = (\mathbf{x}_{\perp 0}, 0)$ and velocity $\mathbf{v}_0$. The gradient map defined by Supplementary Equation 28 does not include explicitly the imaging magnification factor $\mathcal{M}$, which is instead incorporated into the scheme via the re-scaled initial flux Ψ_0. Although Supplementary Equation 26 is non-linear, it has a unique solution for $\nabla\phi$ given any (positive) flux distribution Ψ with a boundary condition

$$\hat{\mathbf{n}} \cdot \nabla\phi\,(\mathbf{x}_{\perp 0}) = \hat{\mathbf{n}} \cdot \mathbf{x}_{\perp 0} \quad \text{on } \delta S, \tag{29}$$

where δS is the boundary of the region containing the flux distribution of interest, and $\hat{\mathbf{n}}$ the normal to that boundary [8].

The flux relation of Supplementary Equation 26 with boundary condition the Supplementary Equation 29 was inverted numerically using a finite-difference scheme applied to the parabolic Monge-Ampère equation, the steady-state solution of which is the solution of the conventional Monge-Ampère equation [9]. In implementing this field-reconstruction algorithm for actual data, two filtering procedures were utilized. First, a Lucy-Richardson deconvolution algorithm was applied to flux images [10, 11]. This is because the strength of path-integrated fields predicted by solving the Monge-Ampère equation is reduced in the case where strong, narrow flux features are affected by finite image resolution ("smearing"). Some of these effects, such as stochastic magnetic diffusion due to small-scale fields, are difficult to eliminate, due to unknown parameters associated with smearing. However, the effect on image resolution due to the finite size of the proton source has been shown to be a convolution of the "unsmeared" flux image resulting from a point proton source with a point-spread function whose precise form is determined by the velocity profile of the protons [7]. In the case of a fusion capsule, this point-spread function has been well-described as Gaussian, with full-width-half-maximum (FWHM) of $50\mathcal{M}\,\mu\mathrm{m} \approx 0.14\,\mathrm{cm}$ when including the imaging-magnification factor [12, 13]. Assuming such a point-spread function for the experimental data, the Lucy-Richardson deconvolution scheme was then implemented. For the particular flux images shown in Figure 4 of the main text and Supplementary Figure 8, ten iterations were found to balance optimally the recovery of the flux distribution with the undesired side effect of Poisson noise enhancement introduced

by the deconvolution process. The efficacy of the deconvolution scheme is further enhanced by the general robustness of the non-linear reconstruction algorithm to small-scale noise [7]. An illustration of successful application of Lucy-Richardson deconvolution for simulated FLASH proton radiography is given later (Supplementary Figure 10).

In addition to the deconvolution, a Gaussian high-pass filter (FWHM $2.2\mathcal{M}l_{\mathrm{i}} \approx 6.16\,\mathrm{cm}$) was applied to a selected flux region, in order to remove long-scale variation in the flux distribution. This procedure is required because, when implemented practically, the initial flux produced by fusion capsules can vary by as much as 50% across a single CR-39 sample, though variations over solid angles $\leq 1.1^\circ$ are typically small compared to the mean flux [14]. Since the dominant structures remaining in the reconstruction are of the order $l_B \sim 300\,\mu\mathrm{m}$, and order-unity flux features are still retained in the filtered flux image, we conclude that our field-reconstruction algorithm captures the energetically dominant magnetic structures.

The flux regions of interest (shown in Figure 4 of the main text between the grids and rods) typically have irregular shapes; to make them compatible with a rectangular finite-difference grid, we embed each section of flux in a larger rectangular region, with the edge filled with uniform mean flux (as calculated from the chosen sample). The initial flux is then chosen to be entirely uniform, with mean value set equal to that of the experimental flux region. The reconstruction algorithm is then applied, with Supplementary Equation 29 specified on the boundary of the larger rectangular region. With the reconstruction completed, an image of the magnitude of the calculated path-integrated field is then re-oriented to the original position of its associated flux region.

The applicability of this scheme to our radiography setup depends on various assumptions: paraxiality, small deflections, point-projection, and radiographic injectivity. Since the distance r_{i} from the proton source to the plasma is much greater than the size ℓ_n of the plasma, we can approximate the section of the beam passing through the plasma as planar, despite the fact that proton beams generated by fusion reactions in a D_2 capsule implosion generally take the form of a uniformly expanding spherical shell [13]. For a given proton, this paraxial approximation is effectively an expansion of the position and velocity of the particle in terms of the paraxial parameter

$$\varphi_0 \equiv \frac{\ell_n}{2r_{\mathrm{i}}} \ll 1\,, \tag{30}$$

the ratio of the size ℓ_n of the region being imaged to the radius r_{i} of curvature. For our experiment, we have $\ell_n \approx 0.06\,\mathrm{cm}$, $r_{\mathrm{i}} \approx 1\,\mathrm{cm}$, giving $\varphi_0 \approx 0.03 \ll 1$, as required. This means that the initial velocity $\mathbf{v}_0$ of a proton with initial position $\mathbf{x}_0$ before interaction with magnetic fields can be written as

$$\mathbf{v}_0 = v_{\mathrm{p}} \left(\hat{\mathbf{z}} + \frac{\mathbf{x}_{\perp 0}}{r_{\mathrm{i}}} \right)\,. \tag{31}$$

Next, typical proton-velocity deflections are assumed small compared to the initial proton velocity. More precisely, it can be shown by integrating the equations of motion under this assumption that a proton with initial position $\mathbf{x}_0$ and velocity $\mathbf{v}_0$ acquires a velocity perturbation

$\mathbf{w}$ due to magnetic forces given by

$$\mathbf{w} \approx \frac{e}{m_\mathrm{p} c v_\mathrm{p}} \mathbf{v}_0 \times \int_0^{l_\mathrm{i}} \mathrm{d}s \, \mathbf{B}(\mathbf{x}(s)) \approx \frac{e}{mc} \hat{\mathbf{z}} \times \int_0^{l_\mathrm{i}} \mathrm{d}s \, \mathbf{B}(\mathbf{x}(s)) \ . \tag{32}$$

The second approximation is obtained by substituting the initial velocity (Supplementary Equation 31) and neglecting $\mathcal{O}(\varphi_0)$ terms. Supplementary Equation 32 implies that the deflection velocity of a proton is perpendicular to its initial direction – that is, $\mathbf{w} \cdot \mathbf{v}_0 \approx \mathbf{w} \cdot \hat{\mathbf{z}} \approx 0$ – and so the deflection angle of the proton is given by

$$\Delta\varphi \equiv \frac{|\mathbf{w}|}{v_\mathrm{p}} \sim \frac{eB\ell_n}{mcv_\mathrm{p}} \, . \tag{33}$$

For our experiment, Figure 4f of the main text shows that the maximum predicted path-integrated magnetic field takes a value of $6\,\mathrm{kG\,cm}$, which in turn gives maximum predicted deflection velocity $w \approx 5.7 \times 10^7\,\mathrm{cm\,s^{-1}}$. For the slowest species of proton used for imaging (3.3 MeV), we have $v_\mathrm{p} \approx 2.5 \times 10^9\,\mathrm{cm\,s^{-1}}$, so $\Delta\varphi \leq 0.02$ for all imaging protons. For magnetic fields varying over shorter length scales, this value is further reduced.

The point-projection assumption – that the distance r_s from the plasma to the screen is much greater than ℓ_n – also holds, meaning that displacements from undeflected proton trajectories acquired inside the plasma due to magnetic forces are negligible compared to the displacements resulting from the free motion of particles after they have exited the plasma, with deflected velocity.

The mapping Supplementary Equation 26 implicitly assumes that no trajectories cross before the protons reach the screen, i.e., that the gradient map (Supplementary Equation 28) is injective. It is not necessarily clear from any particular image whether or not it is injective, an issue discussed at length in [7]. Non-injective images can occur in radiographic setups, and are typically characterized by strong flux features known as caustics, which can be hard to distinguish from strong yet injective flux structures in the presence of effects limiting spatial resolution (smearing effects), such as finite proton-source size. Applying a field-reconstruction algorithm to an image formed by a non-injective gradient map can lead to inaccurately inferred magnetic field morphologies, and underestimates of field strengths [7]. Indeed, the solution of the Monge-Ampère equation is known to provide a lower bound on the RMS of all path-integrated field strengths that can generate a particular flux distribution [8]. However, we claim that for the 15.0 MeV proton images from our experiment, non-injectivity of the gradient map would not significantly alter the reconstructed path-integrated field. The justification of this claim is given in subsequent paragraphs.

An alternative test of the field reconstruction algorithm can be carried out by generating an artificial proton flux image, with the deflections of simulated protons determined by the reconstructed path-integrated field combined with Supplementary Equation 32 (see Supplementary Figure 7a). The test consists in comparing these synthetic images to the experimental ones. The result is that they agree fairly well, although the strength of the strong, narrow flux features in the predicted flux image is reduced compared to the experimental one. Enhancing the

path-integrated field by 20% recovers the observed strength of flux features (see Supplementary Figure 7b), suggesting that the reconstructed path-integrated field as shown in Figure 4f of the main text is marginally underestimated.

The imploding capsule also produces $3.3\,\mathrm{MeV}$ protons from DD reactions, with associated smaller velocity $v \approx 2.5 \times 10^9$ cm s^{-1}. These slower protons are recorded independently from the faster, 15.0 MeV protons; the DD equivalents of the D^{3}He images (displayed in Figure 4 of the main text) are shown in Supplementary Figure 8. For both a single grid experiment (Supplementary Figure 8a), and at early times in a double grid experiment (Supplementary Figure 8b) these 3.3 MeV proton images show relative uniformity, placing an upper bound on the strength of the magnetic field. At later times (Supplementary Figure 8, panels c and d), similar order-unity flux structures can be seen in the 3.3 MeV proton images, as expected from the inverse velocity dependence of the small-deflection angle criterion, Supplementary Equation 33. The similar positioning of these flux structures to structures present in 15.0 MeV proton images is intuitively less obvious, but matches previous findings both for simple analytic cases and other experiments [15]. The increased thickness of the structures in Supplementary Figure 8 compared to those in the 15.0 MeV flux images is consistent with the existence of caustics in the former. As a result, applying the field-reconstruction algorithm to the 3.3 MeV images gives path-integrated fields (Supplementary Figure 8, panels e-h)that underestimate field strength compared to those recovered from the 15.0 MeV images.

Despite the presence of caustics, the 3.3 MeV proton radiographs can be used to verify the assumption of injectivity of the 15.0 MeV images. The turnover time of the plasma motions ($L/u_L \sim 6\,\mathrm{ns}$) is much greater than the difference in transit time of the two species of protons used for imaging ($\leq 0.2\,\mathrm{ns}$). Therefore, to a good approximation, the 3.3 MeV and 15.0 MeV protons are deflected by the same magnetic fields. As a result, any path-integrated field reconstructed from the flux image of one proton species should be consistent with flux images obtained for the other species. This can be tested by generating a predicted image using a reconstructed field from one proton species combined with mapping (Supplementary Equation 28), but varying the initial proton speed to match that of the other proton species. When such a test is carried out for the 15.0 MeV proton reconstructions, the morphology of predicted 3.3 MeV proton images agrees well with the actual 3.3 MeV proton images – see Supplementary Figure 7c for example. Further, numerical experiments investigating the use of our reconstruction algorithm in the regime where caustic appearance cannot be detected by the test given above suggest that the predicted path-integrated field morphology remains relatively robust, though field strength can be somewhat underestimated [7]. This is consistent with the observation (see Supplementary Figures 7b and 7d) that a 20% increase in field strength still leads to similar flux features.

The spectrum of magnetic field energy $E_B(k)$ can be calculated directly from the path-integrated magnetic field if the field's statistics are assumed isotropic [7]. Specifically, the experimental spectrum shown in Figure 4g of the main text and the simulated spectra shown in

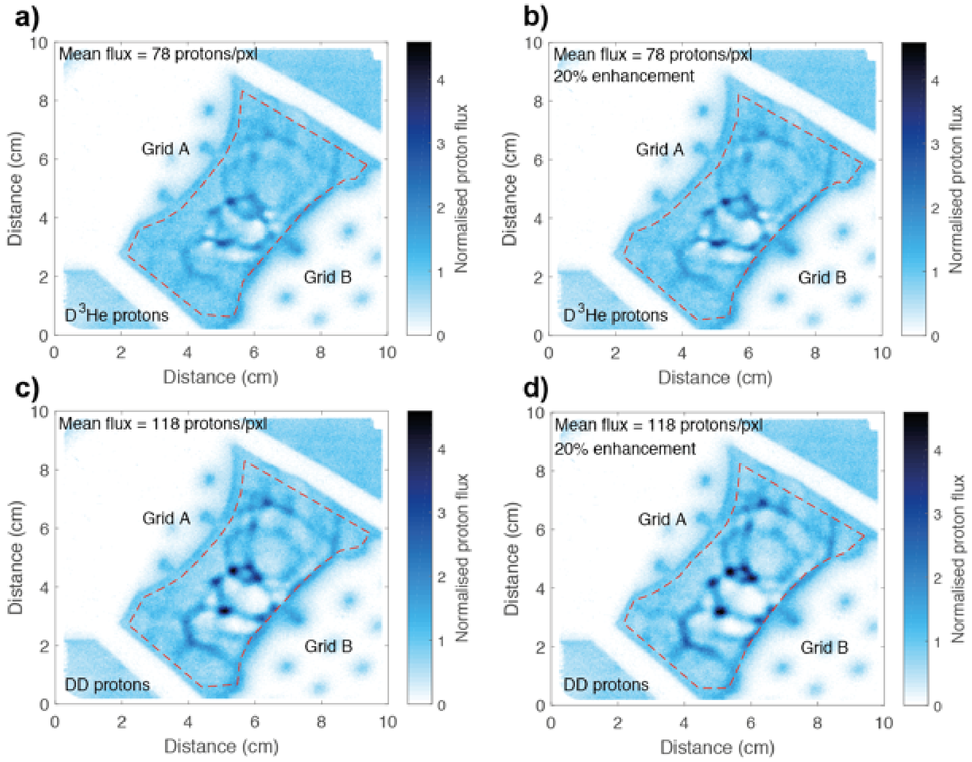

Supplementary Figure 7: **Validation of field reconstruction algorithm.** (a) 15.0-MeV flux image predicted using reconstructed path-integrated magnetic field shown in Figure 4f of the main text. The image is created by seeding uniformly test protons in the region over which the path-integrated field is defined (marked by a dashed red line), before assigning velocities to those particles. These velocities are given by the combination of a particle's initial velocity (Supplementary Equation 31) before interacting with fields, and velocity perturbation (Supplementary Equation 32) due to magnetic fields. The particles are then allowed to propagate to the screen, with dimensions set equal to those in the experiment. The mean flux in each region is set equal to the mean flux of the experimental image. To account for finite source size, a Gaussian point spread function with FWHM 50 $\mathcal{M}\,\mu\mathrm{m} \approx 0.14\,\mathrm{cm}$ is subsequently applied to the images. (b) Same as (a), but with the strength of the path-integrated field increased by 20%. (c) 3.3-MeV flux image predicted using reconstructed path-integrated magnetic field shown in Figure 4f of the main text (cf. Supplementary Figure 8d). The path-integrated field experienced by the slower proton species is the same, but their initial perpendicular velocity is reduced, leading to larger deflections. (d) Same as (a), but with the strength of the path-integrated field increased by 20%.

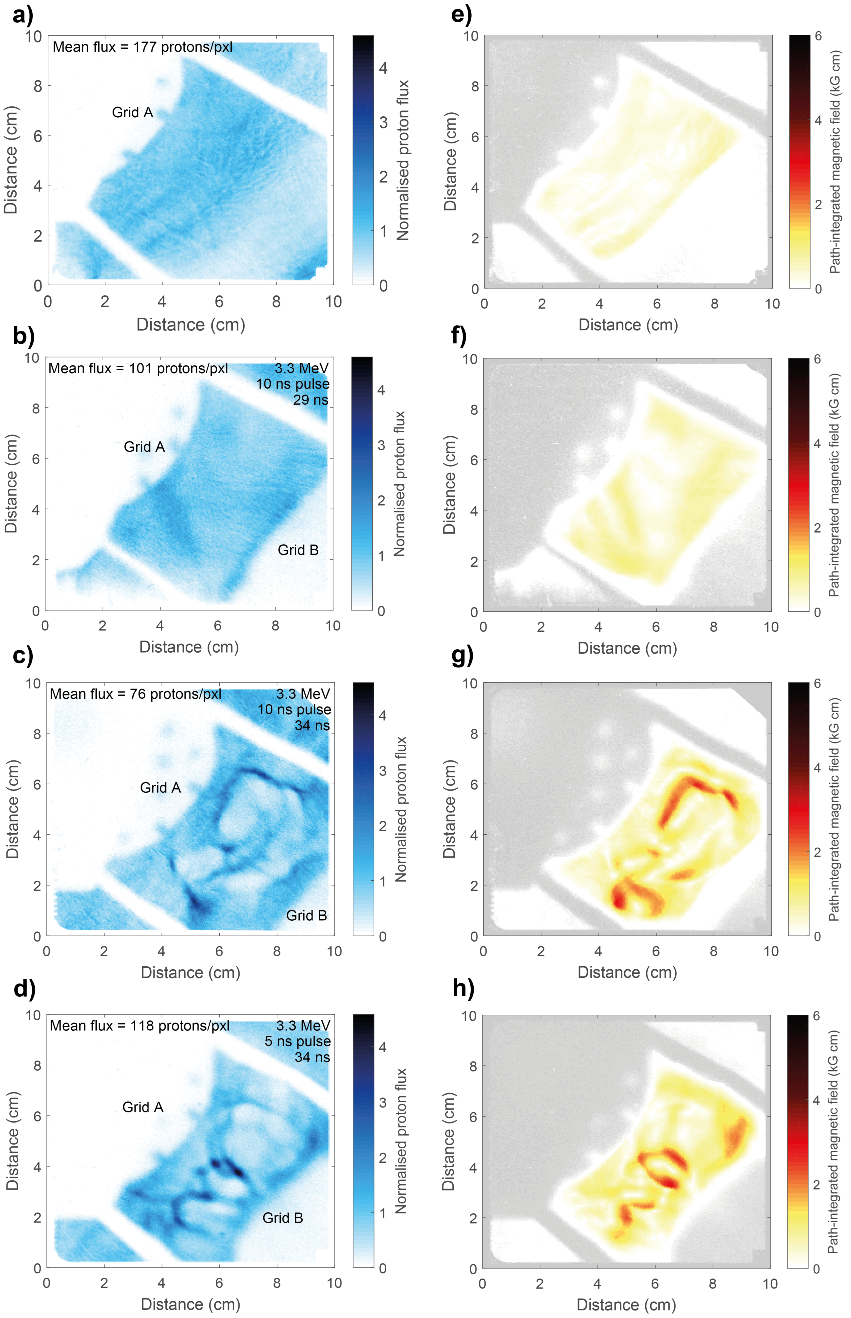

Supplementary Figure 8: **Lower-energy proton radiography.** Radiographs displaying DD 3.3 MeV proton flux as recorded on the CR-39 plate. (a) Capsule implosion at $t = 22$ ns, with only one foil, driven using a 10 ns laser pulse. (b) Capsule implosion at $t = 29$ ns, with both foils, driven with a 10 ns laser pulse. (c) Same as (b) but at $t = 34$ ns. (d) Same as (c) but driven with a 5 ns pulse.

Supplementary Figure 10 are calculated using

$$E_B(k) = \frac{1}{4\pi^2 \ell_n} k E_{\rm path}(k) \ , \tag{34}$$

where $E_{\rm path}(k)$ is the one-dimensional spectrum of the path-integrated magnetic field. The RMS value of the magnetic field can then be evaluated by integrating over all wavenumbers to give

$$B_{\rm rms}^2 = 8\pi \int_0^\infty E_B(k)\, {\rm d}k \, . \tag{35}$$

In practice, the values of the RMS field strength given in the main paper are calculated by summing over pixel values of a Fourier-transformed sample of zero-mean path-integrated magnetic field (the region denoted by a dashed curve in Figure 4f of the main text). Prior to the jet collision, the electron-density scale length is larger, of order the grid's lateral dimensions $\ell_n \geq 3\,{\rm mm}$ (we know this from FLASH simulations). However, as the collision occurs, and the turbulent jet-interaction region forms, the density scale increases to $\ell_n \sim 0.6\,{\rm mm}$, as stated in the main text.

We have also noted in the main text that the magnetic energy spectrum derived from the experimental proton radiography data using Supplementary Equation 34 is suppressed at small wavelengths ($\lesssim 100\ \mu$m). We emphasize that this may not be indicative of the true spectrum, which could follow a flatter power law – yet this would not be reproduced using the reconstruction method. In essence, this is because the injectivity assumption required above for the reconstruction to be valid is scale-dependent. Small-scale magnetic structures are more likely to lead to the crossing of trajectories of neighboring protons than larger structures of the same field strength, even though the deflections associated with the small structures are smaller. For multi-scale stochastic magnetic fields, this means that the reconstruction algorithm applied to small-scale flux structures will produce an underestimate of small-scale path-integrated fields – which in turns leads to the suppression of small wavelengths in the magnetic energy spectrum. In the limit of very small structures, this lack of injectivity manifests itself diffusively, effectively leading to an additional smearing effect. This phenomenon is demonstrated below for the stochastic magnetic fields produced in the FLASH simulation.

Supplementary Method 5: The Collisional Approximation

Starting with the measured values for flow velocities, temperatures, magnetic fields, and densities as given by various experimental diagnostics, we can fully characterize the plasma state. The calculated Debye length is $\lambda_{\rm D} \approx 6.8 \times 10^{-7}$ cm, which is larger than the ion separation length $n_{\rm i}^{-1/3} \approx 3.5 \times 10^{-7}\,{\rm cm}$: this indicates that the average number of particles in the Debye sphere is large, and hence the plasma can be described classically. The Coulomb logarithm is found to be $\log \Lambda \approx 7$.

Assuming an underlying Maxwellian distribution, the ion-ion mean free path is $\lambda_{\rm ii} \approx 1\,\mu{\rm m}$, which (as would be expected for a classical plasma) is larger than the Debye length, but smaller

Quantity	Formula	Value
Average atomic weight (M)	C (49.9%) H (43.8%) Cl (6%)	8.5 a.m.u.
Average ion charge (Z)	C (49.9%) H (43.8%) Cl (6%)	4.4
Temperature after collision ($T_\mathrm{e} = T_\mathrm{i}$)		~ 450 eV
Electron density (n_e)		10^{20} cm^{-3}
Outer scale (L)		0.06 cm
Turbulent velocity (u_L)		100 km s^{-1}
Magnetic field (B)		120 kG
Coulomb logarithm ($\log\Lambda$)	$23.5 - \log n_\mathrm{e}^{1/2} T_\mathrm{e}^{-5/4} - \sqrt{10^{-5} + \frac{(\log T_\mathrm{e} - 2)^2}{16}}$	~ 7
Debye Length (λ_D)	$7.43 \times 10^2 \, \frac{T^{1/2}}{(n_\mathrm{e}(1+Z))^{1/2}}$	6.8×10^{-7} cm
Sound speed (c_s)	$9.80 \times 10^5 \, \frac{[ZT_\mathrm{e} + (5/3)T_\mathrm{i}]^{1/2}}{M^{1/2}}$	1.8×10^7 cm s^{-1}
Mach number	u_L/c_s	$\lesssim 1$
Plasma β	$4.0 \times 10^{-11} \, \frac{n_e T}{B^2}$	125
Ion-ion mean free path (λ_ii)	$2.88 \times 10^{13} \, \frac{T_\mathrm{i}^2}{Z^4 n_\mathrm{i} \log\Lambda}$	9.8×10^{-5} cm
Electron-ion equilibration time (τ_ie)	$3.2 \times 10^8 \, \frac{M T_\mathrm{e}^{3/2}}{Z n_\mathrm{e} \log\Lambda}$	8.4×10^{-9} s
Electron Larmor radius (ρ_e)	$2.38 \, \frac{T_\mathrm{e}^{1/2}}{B}$	4.2×10^{-4} cm
Ion Larmor radius (ρ_i)	$1.02 \times 10^2 \, \frac{M^{1/2} T_\mathrm{i}^{1/2}}{ZB}$	1.2×10^{-2} cm
Viscosity (ν)	$1.92 \times 10^{19} \, \frac{T_\mathrm{i}^{5/2}}{M^{1/2} Z^4 n_\mathrm{i} \log\Lambda}$	4.7×10^2 cm^2 s^{-1}
Fluid Reynolds number (Re)	$u_L L/\nu$	~ 1200
Viscous dissipation scale (l_ν)	$L/Re^{3/4}$	2.8×10^{-4} cm
Resistivity (μ)	$3.2 \times 10^5 \, \frac{Z \log\Lambda}{T_\mathrm{e}^{3/2}}$	1.0×10^3 cm^2 s^{-1}
Magnetic Reynolds number (Rm)	$u_L L/\mu$	~ 600
Resistive dissipation scale (l_μ)	$L/Rm^{3/4}$	4.9×10^{-4} cm
Magnetic Prandtl number (Pm)	Rm/Re	< 1

Supplementary Table 1: Summary of relevant plasma parameters related to the experiment. The units system used for all physical quantities in the above formulas is Gaussian CGS, except temperature expressed in eV.

than all relevant hydrodynamic length scales. This would seem to imply that the plasma is collisional. However, we also need to account for the fact that the plasma formation of the central interaction region is due to two counter-propagating streams. This configuration [16] can lead to kinetic effects such as the Weibel instability that can generate and amplify magnetic fields, as has been observed in laboratory experiments [17, 18]. However, the Weibel instability can only occur in a collisionless or weakly collisional plasma, since collisions strongly suppress its growth rate [19].

To assess the role of the Weibel instability in our experiments, it is helpful to consider three temporal phases: (i) what happens in the two plasma flows before they collide; (ii) what happens when the two plasma flows initially collide, creating a region where counter-propagating flows exist; (iii) and what happens after the interaction region forms.

After their formation, the two plasma flows are strongly collisional and, before they collide, there is no region where counter-propagating flows exist. Therefore the Weibel instability does not occur during phase (i).

Counter-propagating flows exist during phase (ii). The characteristic length scale of the Weibel instability is $\ell_{\mathrm{EM}} \sim c/\omega_{\mathrm{pi}}$, the ion inertial length, where ω_{pi} is the ion plasma frequency. If the mean free path for carbon ions (which are the majority of the high-Z ions in the plasma) is smaller than ℓ_{EM}, carbon ion-ion collisions will suppress the Weibel instability.

To assess the role of the Weibel instability during phase (ii), we use the plasma parameters for the single jet case ($T_{\mathrm{e}} \sim T_{\mathrm{i}} \sim 220\,\mathrm{eV}$, $U \sim 2 \times 10^7\,\mathrm{cm\,s^{-1}}$) measured using Thomson scattering for each of the two counter-propagating plasma flows (see earlier discussion on Thomson scattering). From the FLASH simulations, we estimate a typical ion density for the flows $n_{\mathrm{i}} \sim 1.1 \times 10^{18}\,\mathrm{cm^{-3}}$. Given the plasma composition, the carbon ion number density is then $n_Z \sim 5.5 \times 10^{17}\,\mathrm{cm^{-3}}$. Using these parameters, the characteristic length of the Weibel instability for carbon ions is:

$$\begin{aligned} \ell_{\mathrm{EM}} &\sim c/\omega_{\mathrm{pi}} \sim 2.28 \times 10^7 Z^{-1} \left(\frac{M}{n_Z}\right)^{\frac{1}{2}} \\ &\sim 180\,\mu\mathrm{m} \left(\frac{Z}{6}\right)^{-1} \left(\frac{M}{12\,\mathrm{a.m.u.}}\right)^{\frac{1}{2}} \left(\frac{n_Z}{5.5 \times 10^{17}\,\mathrm{cm^{-3}}}\right)^{-\frac{1}{2}} . \end{aligned} \tag{36}$$

By comparison, the ion-ion mean free path for carbon collisions when the temperature of the colliding flows is much smaller than the ion energy due to bulk flow velocity [20–23] is:

$$\begin{aligned} \ell_{\mathrm{mfp}} &\sim 5 \times 10^{-13} \frac{M^2 U^4}{Z^4 n_Z} \\ &\sim 160\,\mu\mathrm{m} \left(\frac{Z}{6}\right)^{-4} \left(\frac{M}{12\,\mathrm{a.m.u.}}\right)^{2} \left(\frac{n_Z}{5.5 \times 10^{17}\,\mathrm{cm^{-3}}}\right)^{-1} \left(\frac{U}{2 \times 10^7\,\mathrm{cm\,s^{-1}}}\right)^{4} . \end{aligned} \tag{37}$$

Since $\ell_{\mathrm{mfp}} < \ell_{\mathrm{EM}}$ we conclude that the Weibel instability is not important during phase (ii).

For phase (iii), we consider two spatial regions: (a) the surface layers of the interaction region that the plasma flows penetrate and (b) the remaining volume of the interaction region.

To assess the role of the Weibel instability in the surface layers of the interaction region that the plasma flows penetrate, we use the plasma parameters for the interaction region given in Supplementary Table 1 ($T_\mathrm{e} \sim T_\mathrm{i} \sim 450\,\mathrm{eV}$, $n_\mathrm{i} \sim 2.3 \times 10^{19}\,\mathrm{cm}^{-3}$). The number density of the carbon ions in the interaction region is then $n_Z \sim 1.2 \times 10^{19}\,\mathrm{cm}^{-3}$ and each plasma flow collides with the interaction region with velocity $U \sim 2 \times 10^7\,\mathrm{cm\,s}^{-1}$. The characteristic length scale of the Weibel instability is then $\ell_\mathrm{EM} \sim 38\,\mu\mathrm{m}$. By comparison, the mean free path for ion-ion collisions is $\ell_\mathrm{mfp} \sim 7\,\mu\mathrm{m}$. Since $\ell_\mathrm{mfp} < \ell_\mathrm{EM}$ the Weibel instability is not important in region (a). The plasma in the remaining volume of the interaction region is strongly collisional, so the Weibel instability is not important in region (b), either.

Thus the Weibel instability is not important during any of the three temporal phases of the experiment.

Since kinetic effects are not important, it is reasonable to describe the developed interaction-region plasma as collisional, with approximately Maxwellian distributions for both electrons and ions. Under this assumption, the temperature equilibration time between ions and electrons is calculated to be $\tau_\mathrm{ie} \approx 8.4\,\mathrm{ns}$, which is consistent with the assumption of a plasma in thermal equilibrium. Following on from this conclusion, we can estimate various transport coefficients. The appropriate form of these depends on the magnetization of the component species in the plasma; the Larmor frequencies for electrons and ions (assuming $B \approx 120\,\mathrm{kG}$) multiplied by their respective collision times are

$$\Omega_\mathrm{i}\tau_\mathrm{ii} \approx 5.8 \times 10^{-2} \ll 1 \qquad \Omega_\mathrm{e}\tau_\mathrm{ie} \approx 2.3 > 1. \tag{38}$$

This implies that the electrons are weakly magnetized, and ions are entirely unmagnetized. The magnetized state of the electrons has various consequences: anisotropization of the thermal and electrical conductivities and the appearance of various terms in the generalized Ohm's law associated with a finite electron Larmor radius. However, since ion flows dominate bulk motion, it might be inferred that the plasma dynamics would be relatively unaffected by this.

Thus, we conclude that conventional resistive MHD provides a reasonable description of the plasma dynamics. Since $T_\mathrm{e} \sim T_\mathrm{i}$, the electrical conductivity is essentially determined by the electrons, and the viscosity by ions [24]. This gives diffusivities

$$\nu \approx 4.7 \times 10^2\ \mathrm{cm}^2\,\mathrm{s}^{-1}, \qquad \mu \approx 1.0 \times 10^3\ \mathrm{cm}^2\,\mathrm{s}^{-1}. \tag{39}$$

A summary of relevant parameters related to the experiment is given in Supplementary Table 1.

Supplementary Method 6: FLASH 3D Simulations

The experimental platform was designed using FLASH radiation-MHD simulations. FLASH is a parallel, multi-physics, adaptive-mesh-refinement (AMR), finite-volume Eulerian code [25, 26]. The code scales well to over a 100,000 processors, and uses a variety of parallelization techniques including domain decomposition, mesh replication, and threading to utilize hardware resources optimally. The code is publicly available (http://flash.uchicago.edu) and allows

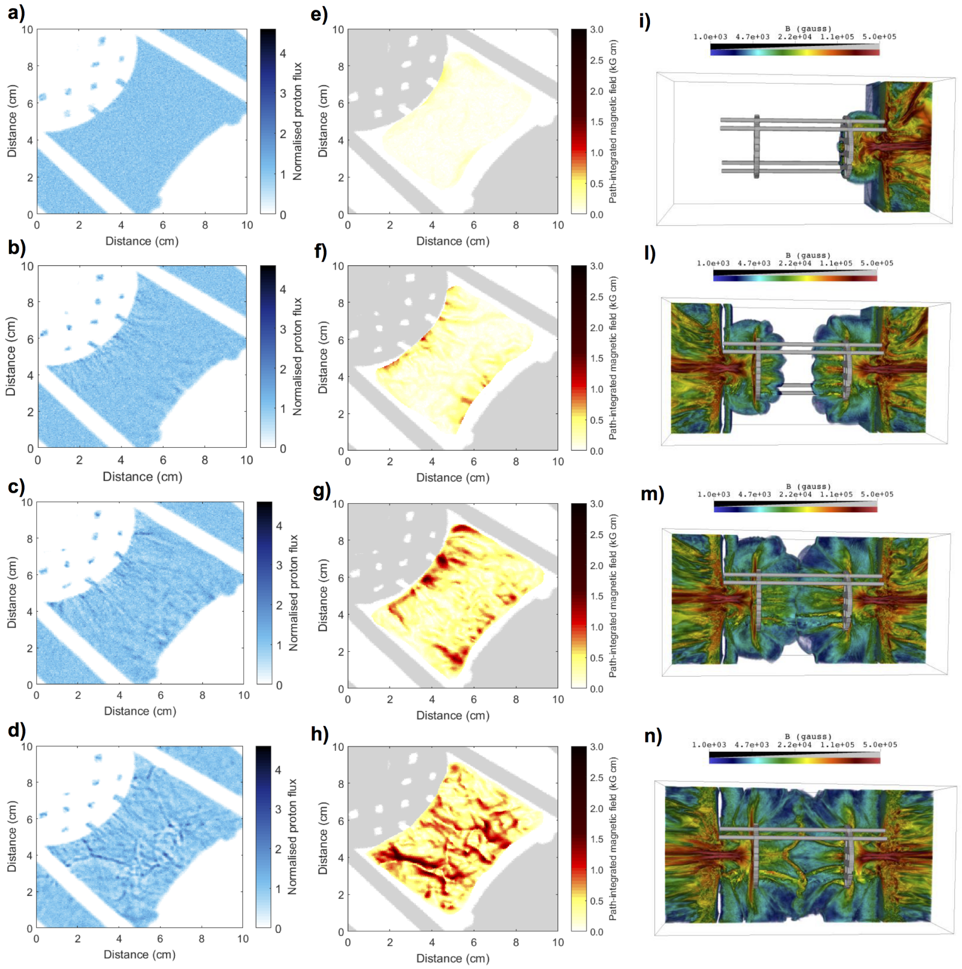

Supplementary Figure 9: **FLASH simulation results.** Simulated proton radiographs (left column) created by passing a proton beam through FLASH-predicted magnetic fields (right column); path-integrated fields reconstructed from the simulated radiographs (middle column). (a) Simulated number of 15 MeV protons detected on a CR-39 plate. The D^3He capsule was imploded at $t = 22$ ns. Fusion reactions occur 0.6 ns after the start of the implosion and the protons are emitted isotropically within a short burst, of ∼150 ps duration. The flight time of the protons to the plasma is 0.1 ns. This particular target had only grid A and a single plasma flow. The chlorinated plastic foil was driven with a 10 ns long pulse shape (see Figure 1 of the main text). (b) Same as (a) but with the capsule imploded at $t = 29$ ns. This target had both our grids and the two chlorinated plastic foils were driven with a 10 ns laser pulse. (c) Same (b), but with the D^3He capsule imploded at $t = 34$ ns. d) Same as (c), but with the chlorinated plastic foils driven with a 5 ns long pulse, which gives higher flow velocities, hence higher magnetic Reynolds numbers and more pronounced structures. (e) Reconstructed path-integrated magnetic fields for case (a). (f) Same for case (b). (g) Same for case (c). (h) Same for case (d). (i) Magnetic field rendering for case (a). (l) Same for case (b). (m) Same for case (c). (n) Same for case (d).

one to model problems in a wide range of disciplines including astrophysics, cosmology, combustion, fluid dynamics, turbulence, and high-energy-density laboratory plasmas (HEDLP).

To design the experiments, we performed an extensive series of moderate-fidelity 2D cylindrical FLASH radiation-MHD simulations on the Beagle 2 cluster at the University of Chicago followed by a smaller set of high-fidelity 3D FLASH radiation-MHD simulations on the Mira supercomputer at the Argonne National Laboratory. This simulation campaign is described in detail in a companion paper [27]. The numerical modeling of the experimental platform employed the entire suite of HEDLP capabilities [27, 28] of the FLASH code: three-temperature (3T) MHD solvers [29], non-ideal MHD effects such as magnetic resistivity [28] and Biermann battery [30, 31], heat exchange between ions and electrons, implicit (using the HYPRE library, https://computation-rnd.llnl.gov/linear_solvers/software.php) thermal conduction and radiation transport in the multi-group diffusion approximation, 3T tabulated equations of state and material opacities, and laser beams that are modeled with geometric-optics ray-tracing and deposit energy via inverse Bremsstrahlung. The FLASH code has a well-established record in modeling turbulent flows, magnetized turbulence, and dynamo action. A number of articles have used the code to simulate compressible and weakly compressible hydrodynamic turbulence [32–35] and turbulent amplification of magnetic fields [36–38].

We have used the FLASH code to simulate the experiment and reproduce the diagnostics results as shown in Figure 4 of the main text. This is illustrated in Supplementary Figure 9. The computational domain spans 0.625 cm in the X and Y directions, and 1.25 cm along Z – the line of centers between the two targets. The spatial resolution is $\sim 25\ \mu$m. Supplementary Figure 9 clearly shows the same trend that is seen in the experimental data: a significant increase of the magnetic field following the collision of the two plasma flows. This is reflected in both the change in morphology of the proton radiographs, and in the path-integrated magnetic fields.

The FLASH code can also be used to investigate the efficacy of some of the techniques employed for analyzing the proton radiographs: in particular, the Lucy-Richardson deconvolution scheme used to correct for a finite proton source size, and the calculation of $B_{\rm rms}$. Synthetic proton radiographs can be generated using the FLASH simulation with both a point source (Supplementary Figure 10c) and a more realistic finite $40\,\mu$m source (Supplementary Figure 10a). This allows the reconstructed path-integrated field from the point source to be compared with the reconstructed field from the finite source, both with and without the use of the deconvolution algorithm (Supplementary Figure 10). Supplementary Figure 10a shows that without the deconvolution algorithm, the reconstruction process leads to lower field strengths. However, Supplementary Figure 10b shows that with the deconvolution algorithm, a much closer match is achieved. This is also reflected in the magnetic-field spectra (Supplementary Figure 10d) predicted from the proton radiography using Supplementary Equation 34.

As mentioned in the main text, FLASH also gives the magnitude of the RMS field generated initially by the laser interaction as $\lesssim 4$ kG. The simulated time history of the magnetic field and its amplification (peak values $B_{\rm max}$ and RMS values $B_{\rm rms}$ in a 500 μm control volume that tracks the plasma front and the interaction region, see also [27]) is given in Supplementary Figure 11b. In order to assess the impact of the Biermann battery mechanism in the amplification

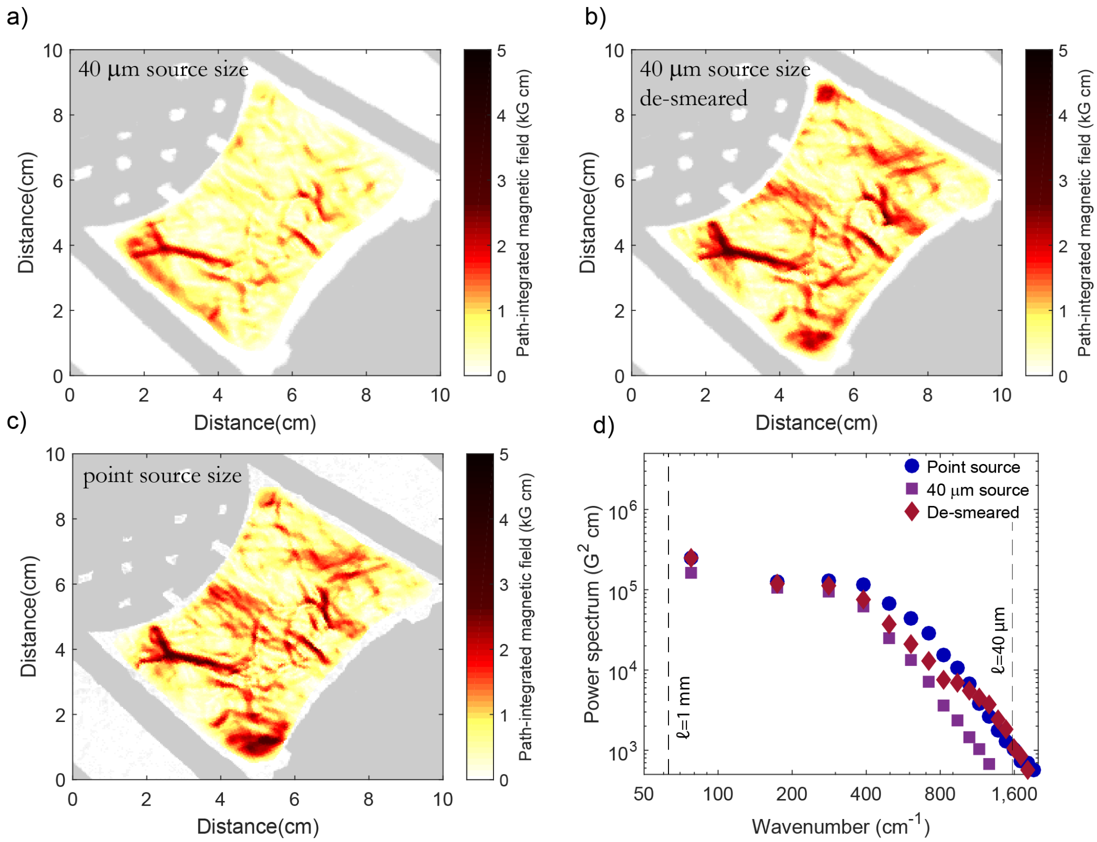

Supplementary Figure 10: **Effect of proton capsule size.** (a) Synthetic reconstruction of the path-integrated magnetic field from simulated proton radiography images creating using a finite-size proton source of diameter 40 μm. (b) Same as (a) but with the Lucy-Richardson deconvolution scheme included in the reconstruction algorithm. (c) Same as (a) but with a point source of 2×10^{-6} μm diameter. d) Extracted magnetic-field spectra for the three previous cases.

process, we repeat our calculations and turn the Biermann battery term off in the interaction region (Supplementary Figure 11a), for times $t > 23$ ns. The resulting time histories (dashed lines in Supplementary Figure 11b) do not show significant difference from those done including the Biermann effect at all times (solid lines in Supplementary Figure 11b). This applies to both peak and RMS values of the magnetic field in the interaction region.

The FLASH magnetic fields can also be used to illustrate the phenomenon described above of suppression of small-wavelength structures when reconstructing magnetic spectra from proton radiographs. Supplementary Figure 11c shows the magnetic energy spectrum derived from the path-integrated field as measured by test protons used for imaging, as well as from the reconstructed path-integrated magnetic field from synthetic proton images of FLASH magnetic fields. The latter in particular shows a much steeper tail than the true FLASH spectrum, with a slope that matches the experimental results of Figure 4g of the main text.

The FLASH simulation results have also been used to recover estimates of the Faraday rotation angle, as in Figure 3c of the main text. To compute the angle, we integrate Supplementary Equation 23 along the complete path of the optical Thomson scattering beam. The latter is discretized into multiple two-segment lines, each of which connect the entry port of the diagnostic laser with a computational cell in the scattering volume, and the computational cell in the scattering volume with the collection lens. The line integrals are subsequently averaged and weighed by the electron density of the cell in which they "scattered," to yield a time-resolved Faraday rotation angle. Supplementary Figure 11d shows the resulting angle (solid blue line) for a 3 ns interval, starting at 32.5 ns as the blue line in Figure 3c of the main text. The simulated rotation angle is qualitatively similar to the experimental result and undergoes sign reversals on comparable timescales. As a consistency check, the simulated RMS angle can also be used in the scaling equation for $B_{\|,\mathrm{rms}}$ in the main text (assuming $n_\mathrm{e} = 10^{20}\,\mathrm{cm}^{-3}$ and $\ell_n \ell_B = 0.2\,\mathrm{mm}^2$ as in the experiment). The resulting RMS magnetic field is in good agreement with the *in situ* measurement of B_rms shown in Supplementary Figure 11b, for the same time interval, corroborating the estimate of $\ell_n \ell_B$. Conversely, the simulated Faraday rotation angle for the single flow case (green solid line in Supplementary Figure 11d) has negligible values, as expected: the seed magnetic fields carried by the plasma flow are too weak to incur a significant change in the polarization angle (see also the green line in Figure 3c of the main text).

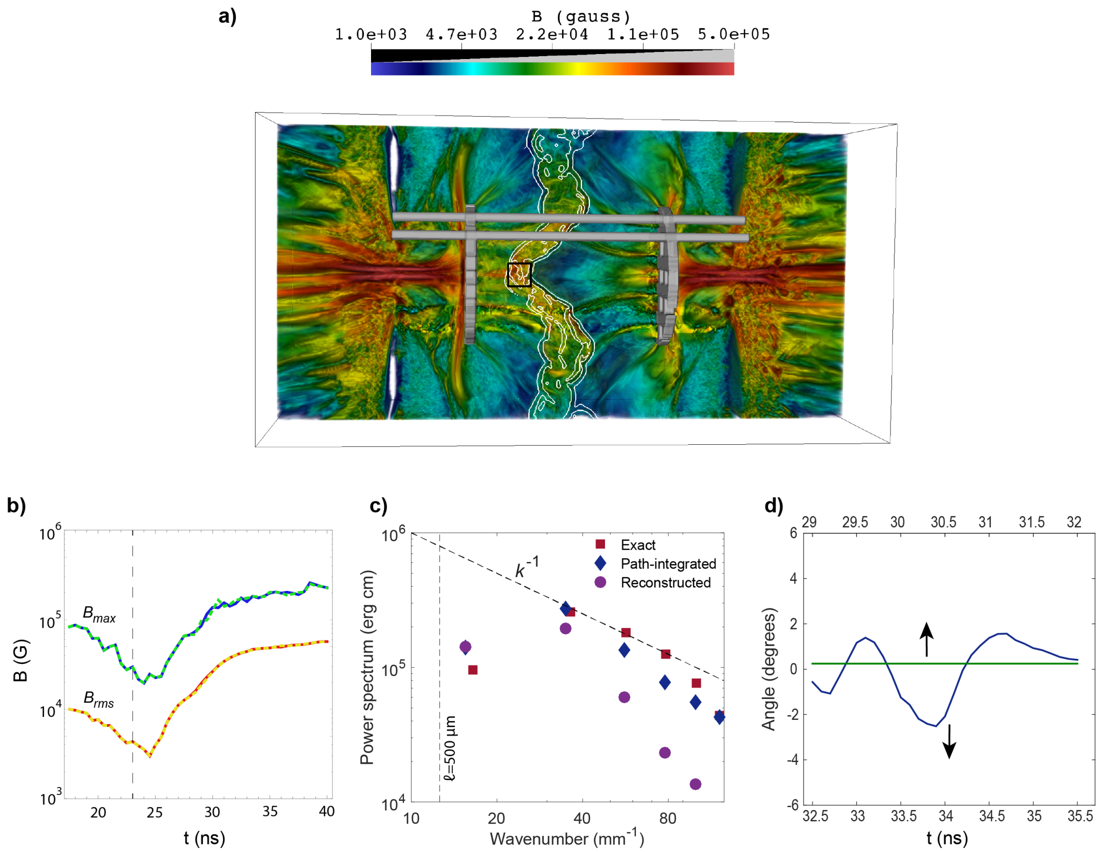

Supplementary Figure 11: **Magnetic field generation in the jet interaction region.** (a) Rendering of the FLASH magnetic field strength at $t = 40$ ns for the 5 ns laser drive. The region where the Biermann battery is turned off is outlined by white boundaries. (b) Evolution of maximum field strength, $B_{\rm max}$, and the RMS field, $B_{\rm rms}$, in the control volume indicated by a square box in panel (a). The plot includes both the results of the full simulation (solid lines), and a simulation where the Biermann battery term was switched off after 23 ns (dashed lines). (c) Magnetic energy spectrum calculated inside the 500 μm control volume using three methods: using FLASH magnetic field values (red squares, see also Figure 2d of the main text); integrating the simulated magnetic field in the control volume along the proton probe path (blue diamonds); and from the reconstructed field from FLASH synthetic proton radiography images (purple circles). (d) Synthetic Faraday rotation angle from the FLASH simulations: the green solid line corresponds to the single flow case when the diagnostic is probing the seed magnetic fields that are advected into the turbulent region. The line is flat and near zero, consistent with weak magnetic fields. The blue solid line corresponds to the colliding flows case, when the diagnostic is probing the magnetic fields after their amplification (see also Figure 3c of the main text). The line exhibits time-variability and Faraday rotation angles of a few degrees, consistent with fluctuating strong magnetic fields.

Supplementary References

[1] Evans DE, Katzenstein J. Laser light scattering in laboratory plasmas. *Rep. Prog. Phys.* **32**, 207–271 (1969).

[2] Arévalo P, Churazov E, Zhuravleva I, Hernández-Monteagudo C, Revnivtsev M. A mexican hat with holes: calculating low-resolution power spectra from data with gaps. *Mon. Not. R. Astron. Soc.* **426**, 1793–1807 (2012).

[3] Churazov E, *et al*. X-ray surface brightness and gas density fluctuations in the coma cluster. *Mon. Not. R. Astron. Soc.* **421**, 1123–1135 (2012).

[4] Cross JE, Reville B, Gregori G. Scaling of magneto-quantum-radiative hydrodynamic equations: from laser-produced plasmas to astrophysics. *Astrophys. J.* **795**, 59 (2014).

[5] Ensslin TA, Vogt C. The magnetic power spectrum in faraday rotation screens. *Astron. Astrophys.* **401**, 835–848 (2003).

[6] Segre S. A review of plasma polarimetry-theory and methods. *Plasma Phys. Contr. Fusion* **41**, R57 (1999).

[7] Bott AFA, *et al*. Proton imaging of stochastic magnetic fields. *J. Plasma Phys.* **83** (2017).

[8] Gangbo W, McCann R. The geometry of optimal transportation. *Acta Math.* **177**, 113–161 (1996).

[9] Sulman M, Williams J, Russell R. An efficient approach for the numerical solution of the monge–ampère equation. *Appl. Numer. Math.* **61**, 298–307 (2011).

[10] Richardson W. Bayesian-based iterative method of image restoration. *J. Opt. Soc. Am.* **62**, 55–59 (1972).

[11] Lucy L. An iterative technique for the rectification of observed distributions. *Astron. J.* **79**, 745–754 (1974).

[12] Séguin F, *et al*. D 3 he-proton emission imaging for inertial-confinement-fusion experiments. *Rev. Sci. Instrum.* **75**, 3520–3525 (2004).

[13] Li C, *et al*. Measuring E and B fields in laser-produced plasmas with monoenergetic proton radiography. *Phys. Rev. Lett.* **97**, 3–6 (2006).

[14] Manuel ME, *et al*. Source characterization and modeling development for monoenergetic-proton radiography experiments on omega. *Rev. Sci. Instrum.* **83**, 063506 (2012).

[15] Kugland N, *et al*. Self-organized electromagnetic field structures in laser-produced counter-streaming plasmas. *Nature Phys.* **8**, 809–812 (2012).

[16] Drake R, Gregori G. Design considerations for unmagnetized collisionless-shock measurements in homologous flows. *Astrophys. J.* **749**, 171 (2012).

[17] Huntington C, *et al*. Observation of magnetic field generation via the weibel instability in interpenetrating plasma flows. *Nature Phys.* **11**, 173–176 (2015).

[18] Park HS, *et al*. Collisionless shock experiments with lasers and observation of weibel instabilities. *Phys. Plasmas* **22**, 056311 (2015).

[19] Ryutov D, Fiuza F, Huntington C, Ross J, Park HS. Collisional effects in the ion weibel instability for two counter-propagating plasma streams. *Phys. Plasmas* **21**, 032701 (2014).

[20] Trubnikov B. Particle interactions in a fully ionized plasma. *Rev. Plasma Phys.* **1**, 105 (1965).

[21] Park HS, *et al*. Studying astrophysical collisionless shocks with counterstreaming plasmas from high power lasers. *High Energ. Dens. Phys.* **8**, 38–45 (2012).

[22] Ross J, *et al*. Characterizing counter-streaming interpenetrating plasmas relevant to astrophysical collisionless shocks. *Phys. Plasmas* **19**, 056501 (2012).

[23] Zakharov YP. Comment on “studying astrophysical collisionless shocks with counterstreaming plasmas from high power lasers”. *High Energ. Dens. Phys.* **8**, 329–330 (2012).

[24] Braginskii S. Transport processes in a plasma. *Rev. Plasma Phys.* **1**, 205 (1965).

[25] Fryxell B, *et al*. FLASH: an adaptive mesh hydrodynamics code for modeling astrophysical thermonuclear flashes. *Astrophys. J. Suppl. Ser.* **131**, 273–334 (2000).

[26] Dubey A, *et al*. Extensible component-based architecture for FLASH, a massively parallel, multiphysics simulation code. *Parallel Comput.* **35**, 512–522 (2009).

[27] Tzeferacos P, *et al*. Numerical modeling of laser-driven experiments aiming to demonstrate magnetic field amplification via turbulent dynamo. *Phys. Plasmas* **24**, 041404 (2017).

[28] Tzeferacos P, *et al*. FLASH MHD simulations of experiments that study shock-generated magnetic fields. *High Energ. Dens. Phys.* **17**, 24–31 (2015).

[29] Lee D. A solution accurate, efficient and stable unsplit staggered mesh scheme for three dimensional magnetohydrodynamics. *J. Comput. Phys.* **243**, 269–292 (2013).

[30] Fatenejad M, *et al*. Modeling HEDLA magnetic field generation experiments on laser facilities. *High Energ. Dens. Phys.* **9**, 172–177 (2013).

[31] Graziani C, *et al*. The Biermann catastrophe in numerical magnetohydrodynamics. *Astrophys. J.* **802**, 43 (2015).

[32] Fisher R, *et al*. Terascale turbulence computation using the FLASH3 application framework on the IBM Blue Gene/L system. *IBM J. Res. Dev.* **52**, 127–136 (2008).

[33] Benzi R, *et al*. Intermittency and universality in fully developed inviscid and weakly compressible turbulent flows. *Phys. Rev. Lett.* **100**, 234503 (2008).

[34] Benzi R, Biferale L, Fisher R, Lamb D, Toschi F. Inertial range eulerian and lagrangian statistics from numerical simulations of isotropic turbulence. *J. Fluid Mech.* **653**, 221–244 (2010).

[35] Radice D, Couch S, Ott C. Implicit large eddy simulations of anisotropic weakly compressible turbulence with application to core-collapse supernovae. *Comput. Astrophys. Cosmol.* **2**, 7 (2015).

[36] Sur S, Schleicher D, Banerjee R, Federrath C, Klessen R. The generation of strong magnetic fields during the formation of the first stars. *Astrophys. J. Lett.* **721**, L134 (2010).

[37] Federrath C, *et al*. Mach number dependence of turbulent magnetic field amplification: solenoidal versus compressive flows. *Phys. Rev. Lett.* **107**, 114504 (2011).

[38] Federrath C, Schober J, Bovino S, Schleicher D. The turbulent dynamo in highly compressible supersonic plasmas. *Astrophys. J. Lett.* **797**, L19 (2014).
